# Supplementary material for: SDCBP/Syntenin-1 stabilizes BACH1 by disassembling the SCFFBXO22–BACH1 complex in triple-negative breast cancer
Source: EMBO J. 2025 Apr 22;44(11):3085–120. doi: 10.1038/s44318-025-00440-1 (PMC12130529; doi:10.1038/s44318-025-00440-1)
Supplement: Supplementary file 1 — Appendix [file 44318_2025_440_MOESM1_ESM.pdf]

APPENDIX FIGURES

Appendix Figure Contents

Appendix Figure	Appendix Legend	Page
Appendix Figure. S1	SDCBP associates with FBXO22 and impairs SCF^{FBXO22}-targeted substrates for K48-linked degradative ubiquitination.	2
Appendix Figure. S2	Identification of *NDUFA4* and *COX6B2 as ETC genes regulated by the SDCBP-BACH1 axis.*	4
Appendix Figure. S3	TCGA data analysis of SDCBP, BACH1, COX6B2, and NDUFA4 MRNA expression in TNBC patients.	7
Appendix Figure. S4	Targeting SDCBP enhances anti-tumor efficiency of the mitochondrial ETC inhibitors and anti-tumor effect of metformin in TNBC cells	9

Appendix Figure S1

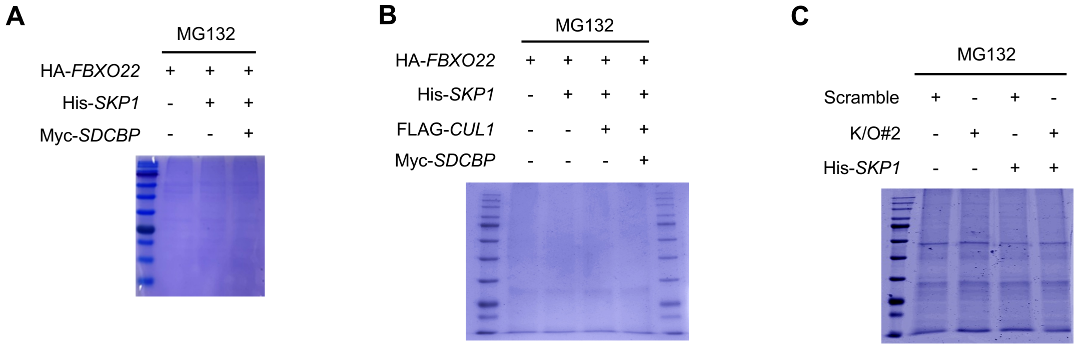

Appendix Figure S1. SDCBP associates with FBXO22 and impairs $\mathrm{SCF}^{\mathrm{FBXO22}}$-targeted substrates for K48-linked degradative ubiquitination.

(A) Coomassie blue-stained SDS-PAGE gel of His-pulldown assay in Fig EV5E. (B) Coomassie blue-stained SDS-PAGE gel of His-pulldown assay in Fig 4D. (C) Coomassie blue-stained SDS-PAGE gel of His-pulldown assay in Fig 4E.

Appendix Figure S2

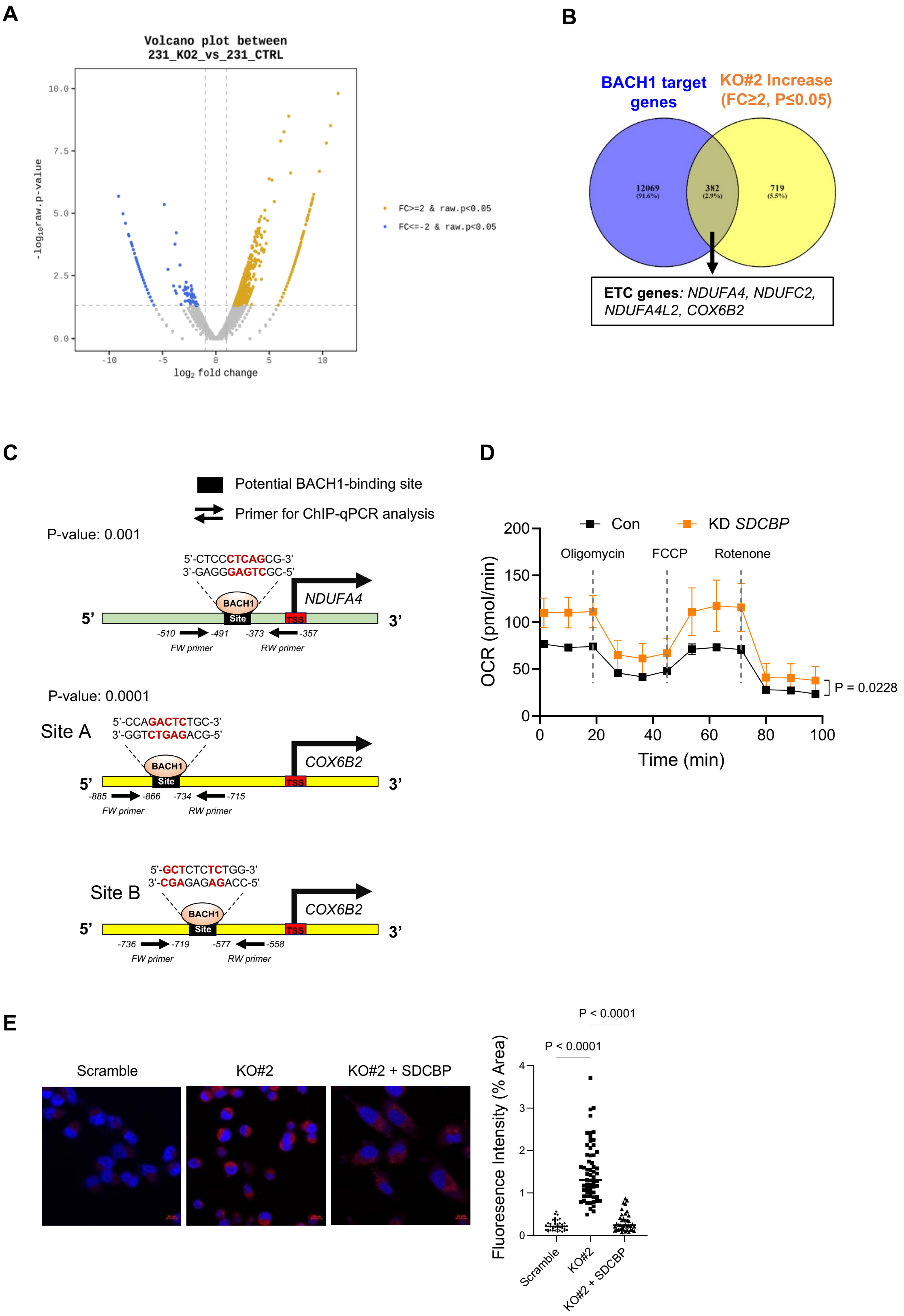

13

Appendix Figure S2. Identification of *NDUFA4* and *COX6B2 as ETC genes regulated by the SDCBP-BACH1 axis.*

(A) RNA sequencing-derived volcano plot identifying the differentially expressed genes (DEGs) in scramble and in SDCBP-KO MDA-MB-231 cells (n=3); The negative-log10 of the *P*-value and the log2 FC were plotted for gene expression in SDCBP-KO relating to the control. Blue and orange-yellow dots indicate downregulated and upregulated DEGs, respectively. Gray dots indicate non-altered genes or non-significant changes. (B) Venn diagram showing the overlap of 382 potential gene candidates identified from the BACH1 target gene library database and upregulated DEGs from RNA-seq. (C) Schematic showing the BACH1-binding sites for approximately 1000 base pairs upstream of the transcriptional start site (TSS) of *NDUFA4* and *COX6B2*. Black arrows indicate primers used for ChIP-qPCR. BACH1-binding sites on the promoters of genes of interest were bioinformatically predicted using the open EPD promoter database with a *P*-value of at least 0.001. (D) Oxygen consumption rate (OCR) showing the mitochondrial activity of MDA-MB-231 cells transfected with scramble siRNA or SDCBP siRNA (n=3). (E) Left, immunofluorescence staining and confocal imaging of the fluorescent signals for TMRE (orange-red color) in the scramble control, SDCBP-KO MDA-MB-231 cells, and SDCBP-KO MDA-MB-231 cells transfected with SDCBP after incubation with TMRE. DAPI (blue color) was used to stain the nucleus. Representative confocal images are shown; scale bars = 20 μm. Right, fluorescent levels of the TMRE were quantified based on their spectral densities (n=36). Data are expressed as the mean ± SEM and analyzed using two-tailed Student's *t* test with Welch's correction (D), or one-way ANOVA (E). All experiments were repeated at least three times unless otherwise indicated. P values less than 0.05 were considered

statistically significant.

Appendix Figure S3

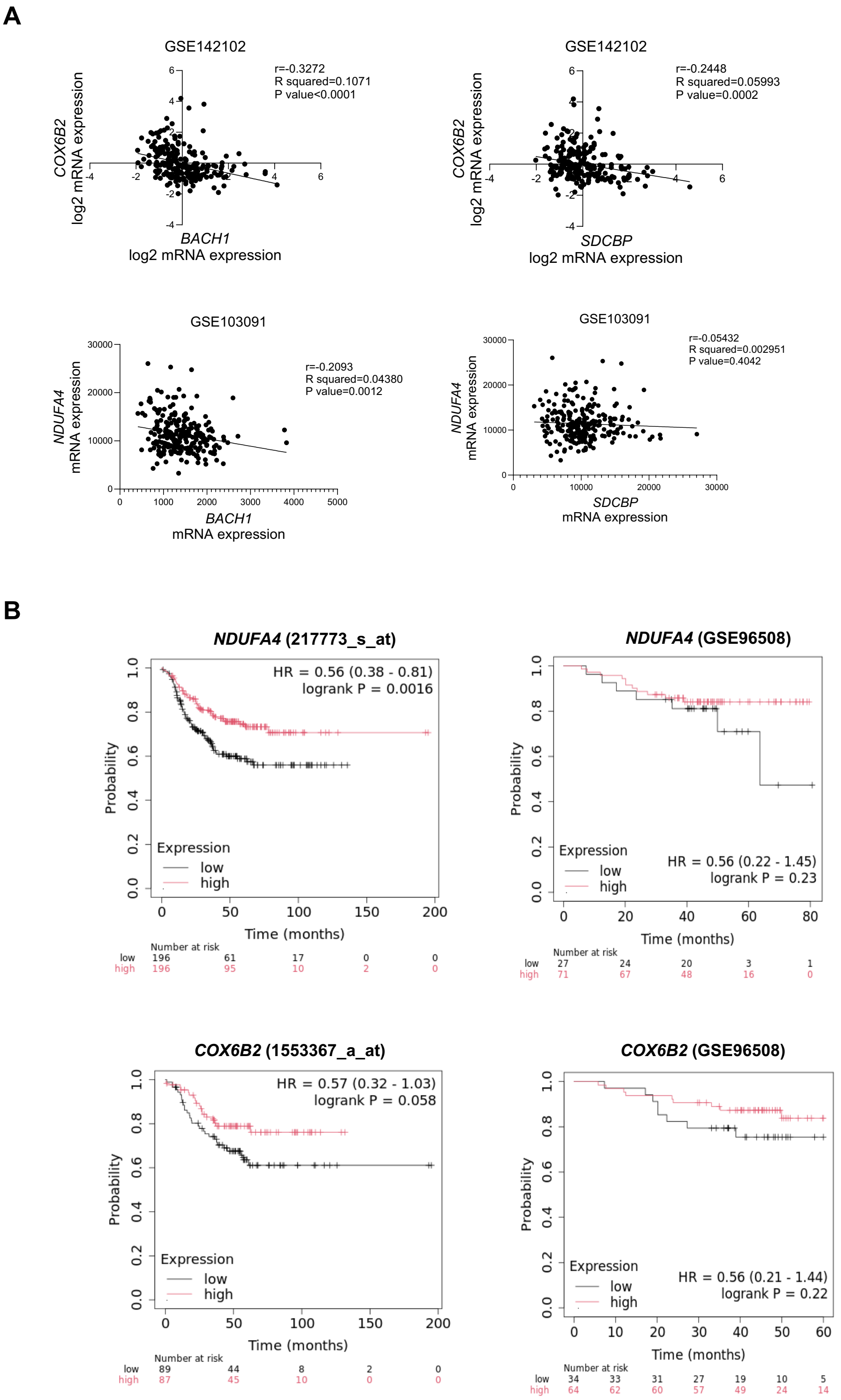

40

Appendix Figure S3. TCGA data analysis of SDCBP, BACH1, COX6B2, and NDUFA4 MRNA expression in TNBC patients.

(A) Upper, TCGA data analysis showing negative correlation between *BACH1* mRNA and *COX6B2* mRNA (Pearson correlation coefficient r = -0.3272, P<0.0001), and between *SCBPB* mRNA and *COX6B2* mRNA (Pearson correlation coefficient r = -0.2448, P=0.0002) in GSE142102 (n = 226) dataset of TNBC patients. Lower, TCGA data analysis showing negative correlation between *BACH1* mRNA and *NDUFA4* mRNA Pearson correlation coefficient = -0.2093, P=0.0012), and between *SCBPB* mRNA and *NDUFA4* mRNA (Pearson correlation coefficient r = -0.05432, P=0.4042) in GSE103091 (n = 238) dataset of TNBC patients. (B) TCGA data analysis showing a negative association between overall survival and *NDUFA4* mRNA and *COX6B2* mRNA expression in TNBC patients.

Appendix Figure S4

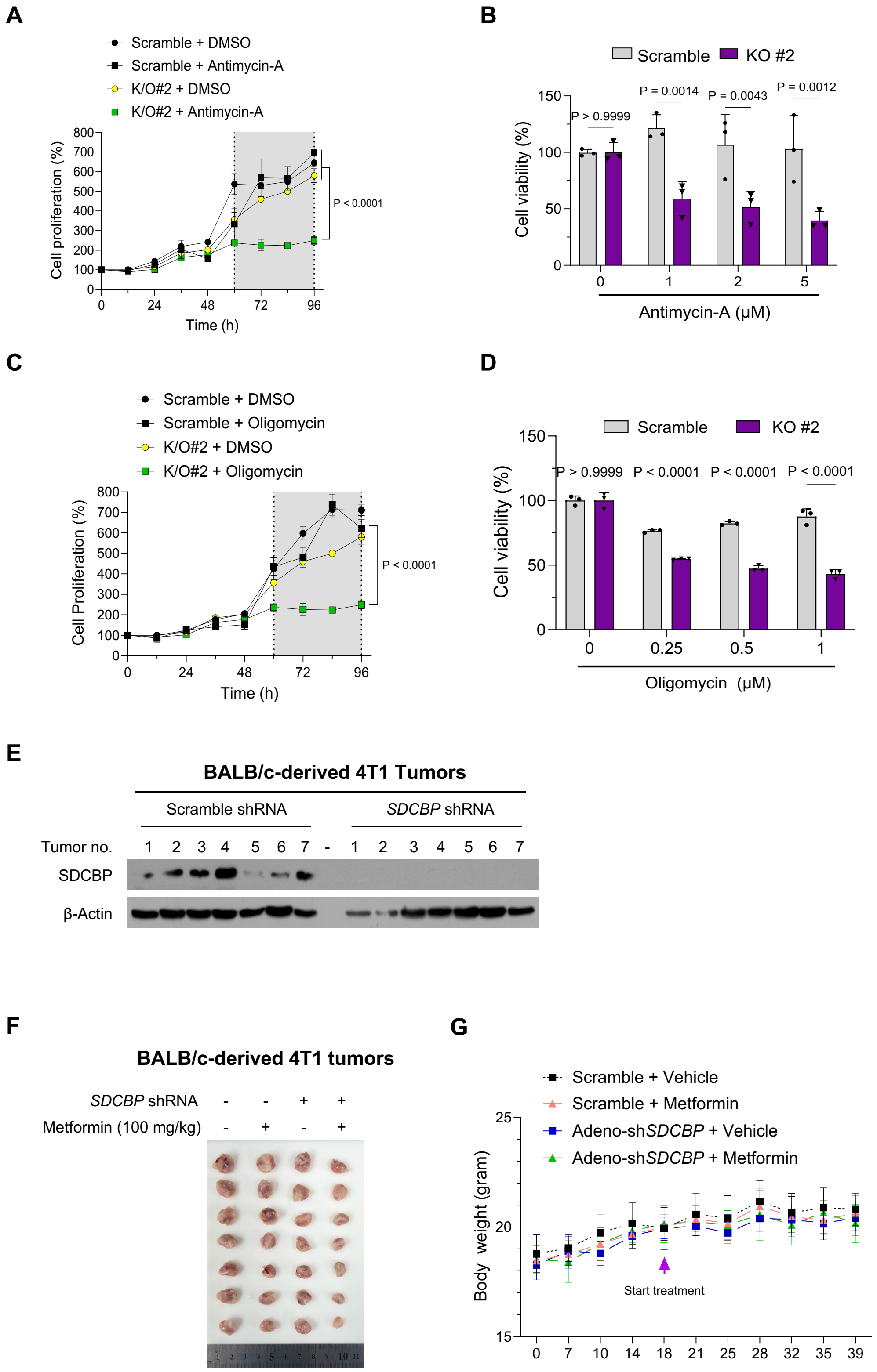

55

Appendix Figure 4. Targeting SDCBP enhances anti-tumor efficiency of the mitochondrial ETC inhibitors and anti-tumor effect of metformin in TNBC cells.

(A) Cell proliferation of the scramble control and SDCBP-KO MDA-MB-231 cells after treatment with antimycin-A for the indicated periods of time. All experiments were performed in triplicate. (B) Cell viability of the scramble control and SDCBP-KO MDA-MB-231 cells after treatment with antimycin-A for 96 h. All experiments were performed in triplicate. (C) Cell proliferation of the scramble control and SDCBP-KO MDA-MB-231 cells after treatment with oligomycin for the indicated periods of time. All experiments were performed in triplicate. (D) Cell viability of the scramble control and SDCBP-KO MDA-MB-231 after treatment with oligomycin for 96 h. All experiments were performed in triplicate. (E) Western blot showing SDCBP protein expression in 4T1 tumors isolated from BALB/c mice at the end of the experiment in Fig 6D. (F) Images of 4T1 tumors isolated from BALB/c mice at the end of the experiment in Fig 6D. (G) Body weight change of 4T1-bearing BALB/c mice during the treatments in Fig 6D (n= 7 mice/group). Data are expressed as the mean ± SEM and analyzed using two-way ANOVA (A, B, C, D). All experiments were repeated at least three times unless otherwise indicated. P values less than 0.05 were considered statistically significant.
