# Supplementary figures and images for: SDCBP/Syntenin-1 stabilizes BACH1 by disassembling the SCFFBXO22–BACH1 complex in triple-negative breast cancer
Source: EMBO J. 2025 Apr 22;44(11):3085–120. doi: 10.1038/s44318-025-00440-1 (PMC12130529; doi:10.1038/s44318-025-00440-1)

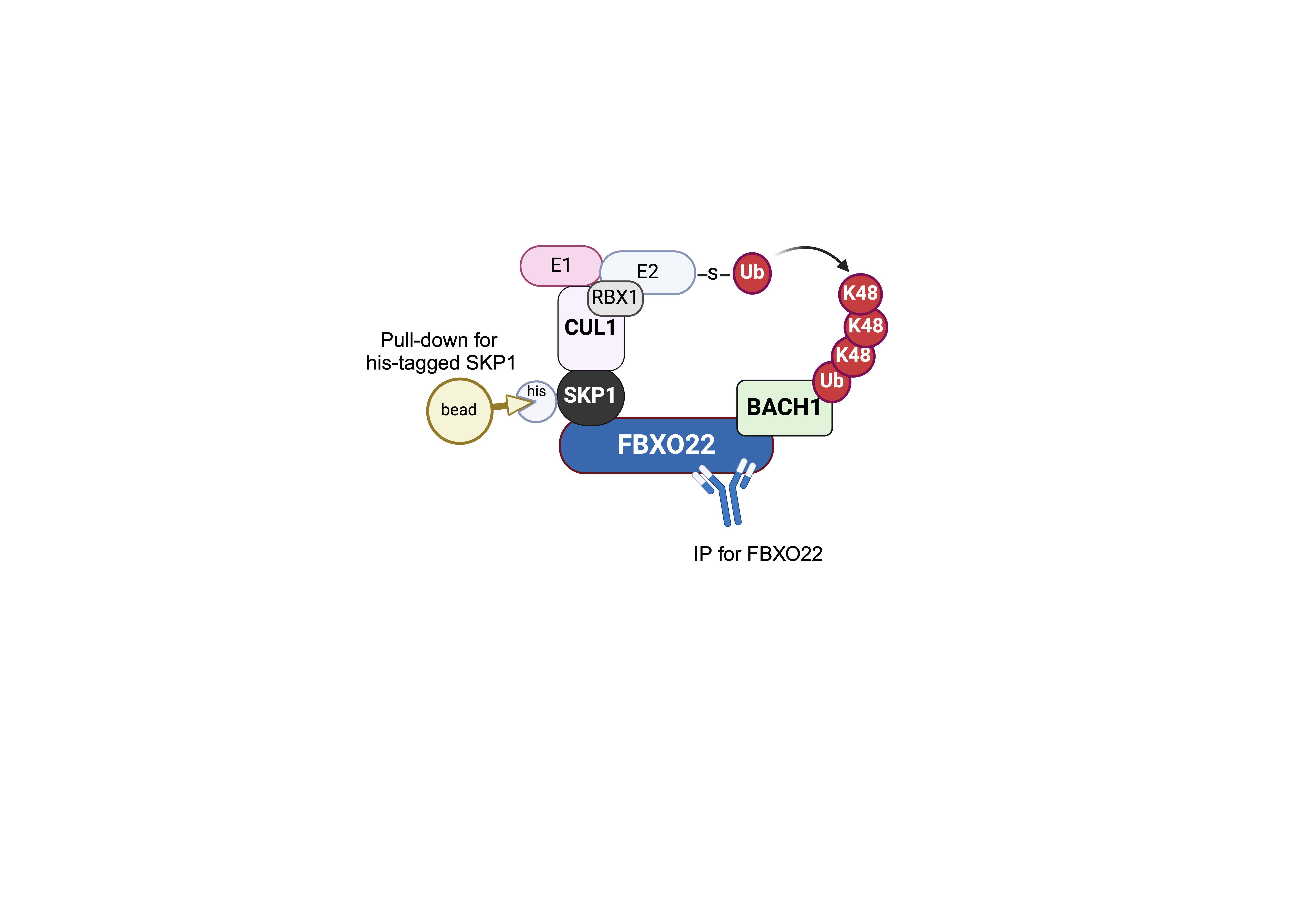

Supplement: Supplementary file 9 — Source data Fig. 7 [file 44318_2025_440_MOESM9_ESM.zip › 20250321_EMBOJ-2024-118617R_Figure 7/EMBOJ-2024-118617R_Synopsis EV Figure 5D.png]

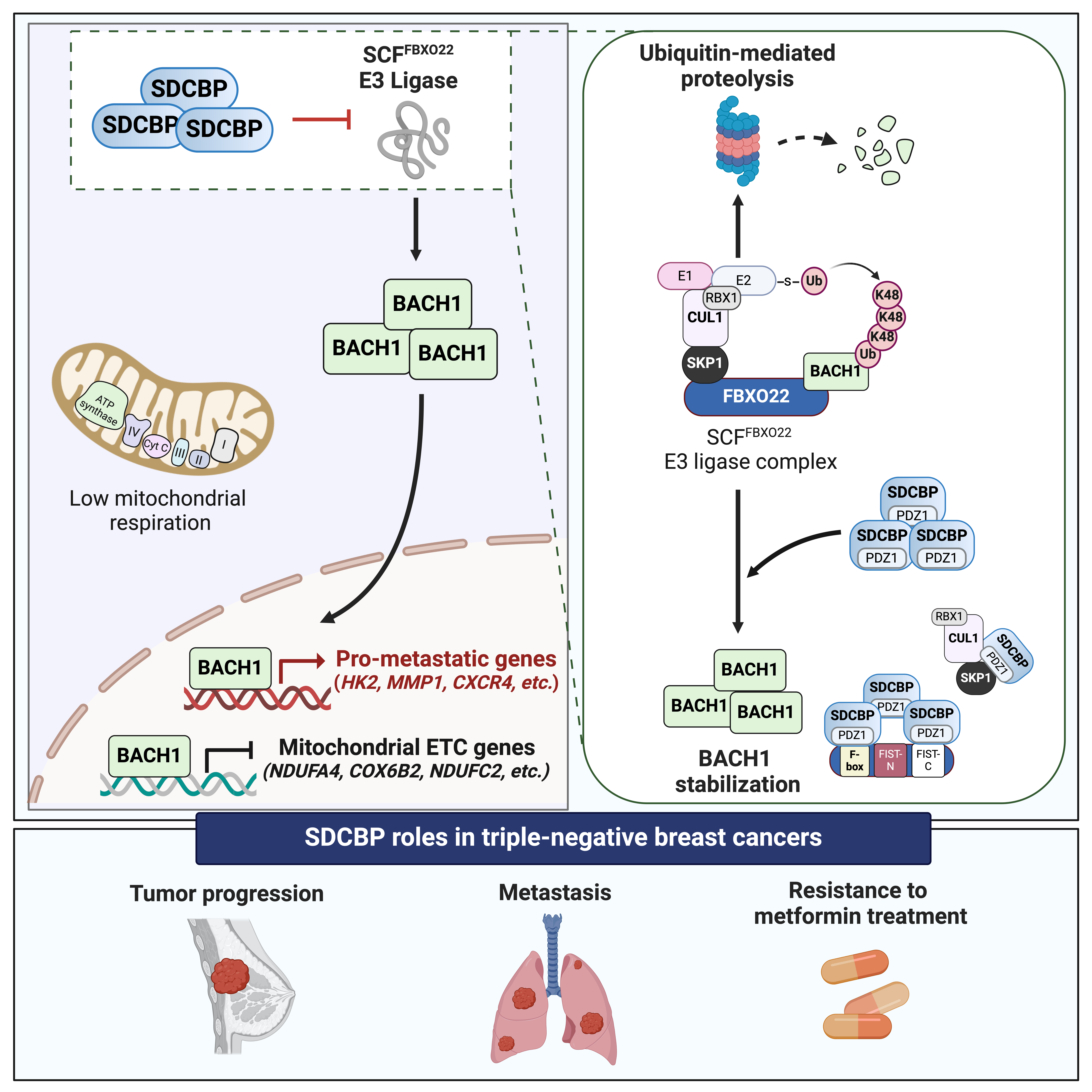

Supplement: Supplementary file 9 — Source data Fig. 7 [file 44318_2025_440_MOESM9_ESM.zip › 20250321_EMBOJ-2024-118617R_Figure 7/EMBOJ-2024-118617_Figure7_ResearchSummary.png]
