# Supplementary material for: SDCBP/Syntenin-1 stabilizes BACH1 by disassembling the SCFFBXO22–BACH1 complex in triple-negative breast cancer
Source: EMBO J. 2025 Apr 22;44(11):3085–120. doi: 10.1038/s44318-025-00440-1 (PMC12130529; doi:10.1038/s44318-025-00440-1)
Supplement: Supplementary file 10 — Expanded View Figures [file 44318_2025_440_MOESM10_ESM.pdf]

Expanded View Figures

Figure EV1. SDCBP regulates the expression of the BACH1 protein and its target genes in TNBC cells.

(**A**) TCGA data analysis showing the correlation between *SDCBP* mRNA and *BACH1* mRNA expression in GSE142102 ($n = 226$) dataset of TNBC patients (Pearson correlation coefficient $r = 0.3245$, $P < 0.0001$). (**B**) TCGA data analysis showing the correlation between *SDCBP* mRNA and *BACH1* mRNA expression in GSE103091 ($n = 238$) dataset of TNBC patients (Pearson correlation coefficient $r = 0.2120$, $P < 0.001$). (**C**) Western blot showing SDCBP, BACH1, and HO-1 protein expression in MDA-MB-231, MDA-MB-468, Hs578T, MCF-7, and T47D cells. (**D**) The expression levels of SDCBP and BACH1 protein in Fig. EV1C were quantified using densitometry and normalized to the housekeeping protein α-tubulin ($n = 3$). (**E**) Real-time qPCR showing *SDCBP* and *BACH1* mRNA expression in MDA-MB-231, MDA-MB-468, Hs578T, MCF-7, and T47D cells ($n = 3$). Quantitative data were normalized to β-actin expression. (**F**) Western blot showing SDCBP and HO-1 protein expression in MDA-MB-231 cells transfected with scramble or BACH1 siRNA. (**G**) Left, western blot showing the protein expression of SDCBP in the scramble and in several SDCBP-KO MDA-MB-231 subclones generated using CRISPR-Cas9 system; Right, real-time qPCR showing the *SDCBP* mRNA expression in scramble and in SDCBP-KO MDA-MB-231 subclones ($n = 3$). (**H**) Real-time qPCR showing the mRNA expression of *BACH1* in MDA-MB-231 cells, in scramble and in SDCBP-KO MDA-MB-231 subclone#2 and subclone#12 ($n = 3$). (**I**) Immunofluorescence staining was used to visualize SDCBP (green color) and BACH1 (red color) in scramble and in SDCBP-KO MDA-MB-231 cells. DAPI (blue color) was used to stain the nucleus ($n = 3$); Representative confocal immunofluorescence images are shown. Scale bar = 20 μm. (**J**) Western blot showing BACH1 and HO-1 protein expression in 4T1 cells infected with scramble or adenoviral SDCBP shRNA. (**K**) Real-time qPCR showing the mRNA expression of BACH1-regulated antioxidant genes (*HMOX1, NQO1*, and *GLCL*) in 4T1 cells transfected with scramble or SDCBP siRNA ($n = 3$); mRNA expression of KEAP1 was the negative control. Data are expressed as the mean ± SEM and analyzed using one-way ANOVA (**D**, **E**, **G**, **H**) or two-way ANOVA (**K**). *P* values less than 0.05 were considered statistically significant. All experiments were repeated at least three times unless otherwise indicated.

© The Author(s)

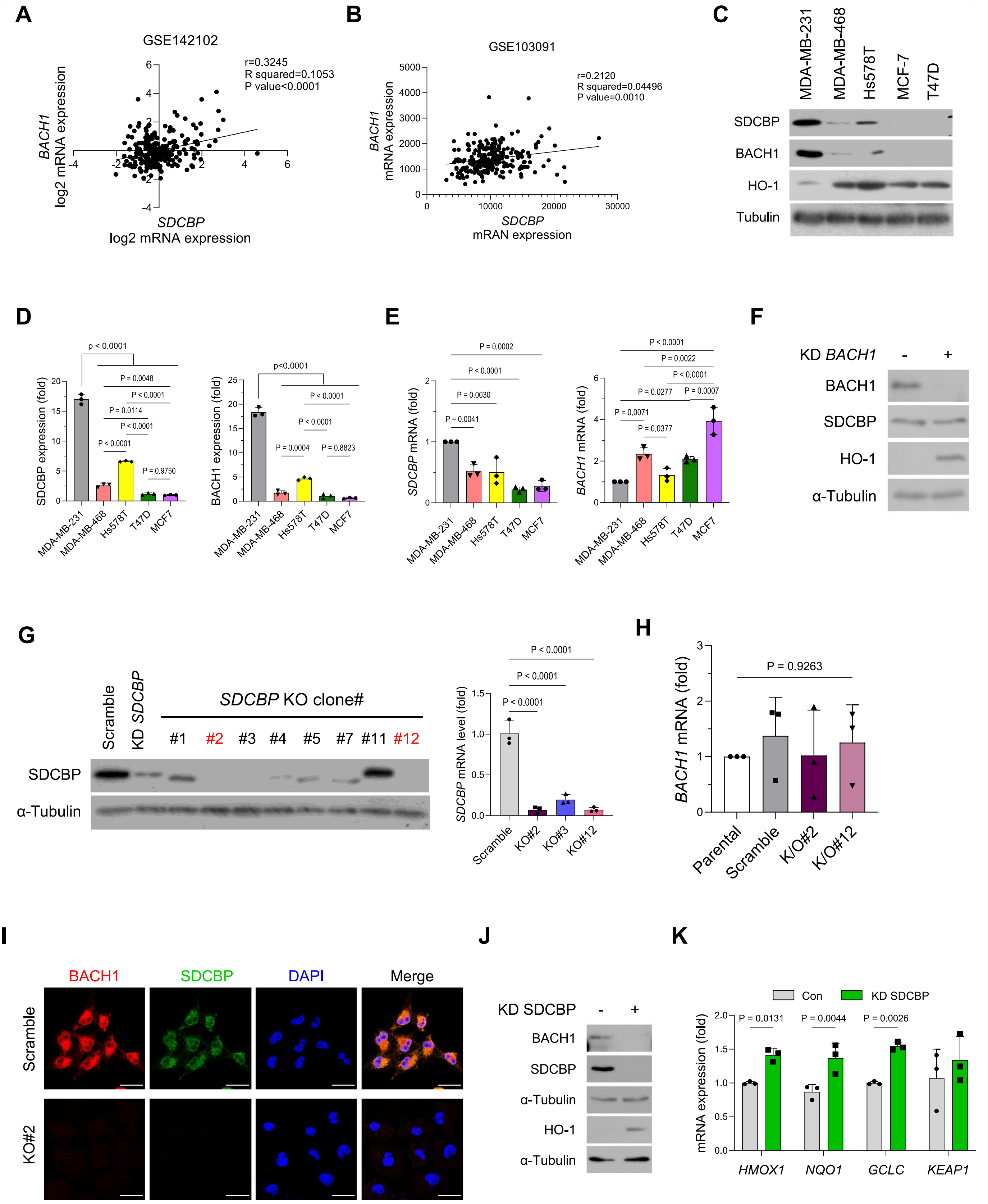
A
GSE142102
r=0.3245
R squared=0.1053
P value<0.0001
BACH1 log2 mRNA expression
SDCBP log2 mRNA expression
B
GSE103091
r=0.2120
R squared=0.04496
P value=0.0010
BACH1 mRNA expression
SDCBP mRAN expression
C
MDA-MB-231
MDA-MB-468
Hs578T
MCF-7
T47D
SDCBP
BACH1
HO-1
Tubulin
D
SDCBP expression (fold)
BACH1 expression (fold)
E
SDCBP mRNA (fold)
BACH1 mRNA (fold)
F
KD BACH1
BACH1
SDCBP
HO-1
α-Tubulin
G
Scramble
KD SDCBP
SDCBP KO clone#
#1 #2 #3 #4 #5 #7 #11 #12
SDCBP
α-Tubulin
SDCBP mRNA level (fold)
Scramble KO#2 KO#3 KO#12
H
BACH1 mRNA (fold)
P = 0.9263
Parental Scramble K/O#2 K/O#12
I
BACH1
SDCBP
DAPI
Merge
Scramble
KO#2
J
KD SDCBP
BACH1
SDCBP
α-Tubulin
HO-1
α-Tubulin
K
Con
KD SDCBP
mRNA expression (fold)
P = 0.0131
P = 0.0044
P = 0.0026
HMOX1 NQO1 GCLC KEAP1

 © The Author(s)

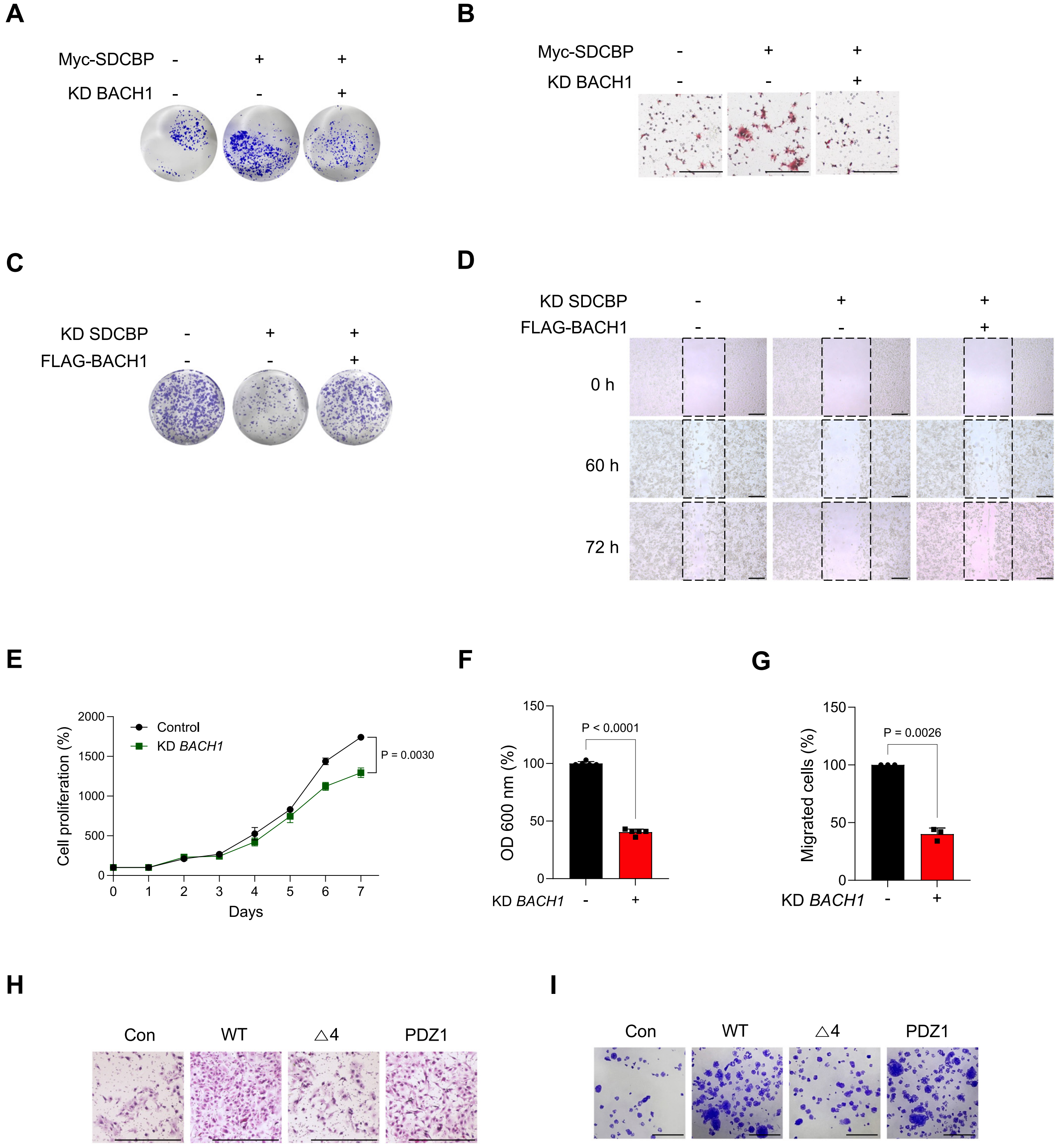
A
Myc-SDCBP - + +
KD BACH1 - - +
B
Myc-SDCBP - + +
KD BACH1 - - +
C
KD SDCBP - + +
FLAG-BACH1 - - +
D
KD SDCBP - + +
FLAG-BACH1 - - +
0 h
60 h
72 h
E
Cell proliferation (%)
Control
KD BACH1
P = 0.0030
Days
F
OD 600 nm (%)
P < 0.0001
KD BACH1 - +
G
Migrated cells (%)
P = 0.0026
KD BACH1 - +
H
Con WT △4 PDZ1
I
Con WT △4 PDZ1

Figure EV2. SDCBP promotes tumor progression by upregulating BACH1 in TNBC cells.

(**A**) Representative images of colony formation in Fig. 2B. (**B**) Representative images of the migrated cells in Fig. 2C. Scale bar = 200 μm. (**C**) Representative images of colony formation in Fig. 2F. (**D**) Representative images of wounding migration in Fig. 2G. Scale bar = 200 μm. (**E**) Cell proliferation of MDA-MB-231 cells transfected with scramble or BACH1 siRNA. Cell proliferation was estimated by an automatic cell counter at the indicated time points ($n = 3$). (**F**) Colony formation of MDA-MB-231 cells transfected with scramble or BACH1 siRNA. The clonogenic ability was assessed and quantified based on the absorbance at 600 nm and normalized to the control ($n = 3$). (**G**) Migration of MDA-MB-231 cells transfected with scramble or BACH1 siRNA. The number of migrated cells were counted and expressed as percentages ($n = 3$). (**H**) Representative images of the migrated cells in Fig. 2K. Scale bar = 500 μm. (**I**) Representative images of colony formation in Fig. 2L. Scale bar = 1000 μm. Data are expressed as the mean ± SEM and analyzed using two-tailed Student's *t* test with Welch's correction (**E**–**G**). *P* values less than 0.05 were considered statistically significant. All experiments were repeated at least three times unless otherwise indicated.

 © The Author(s)

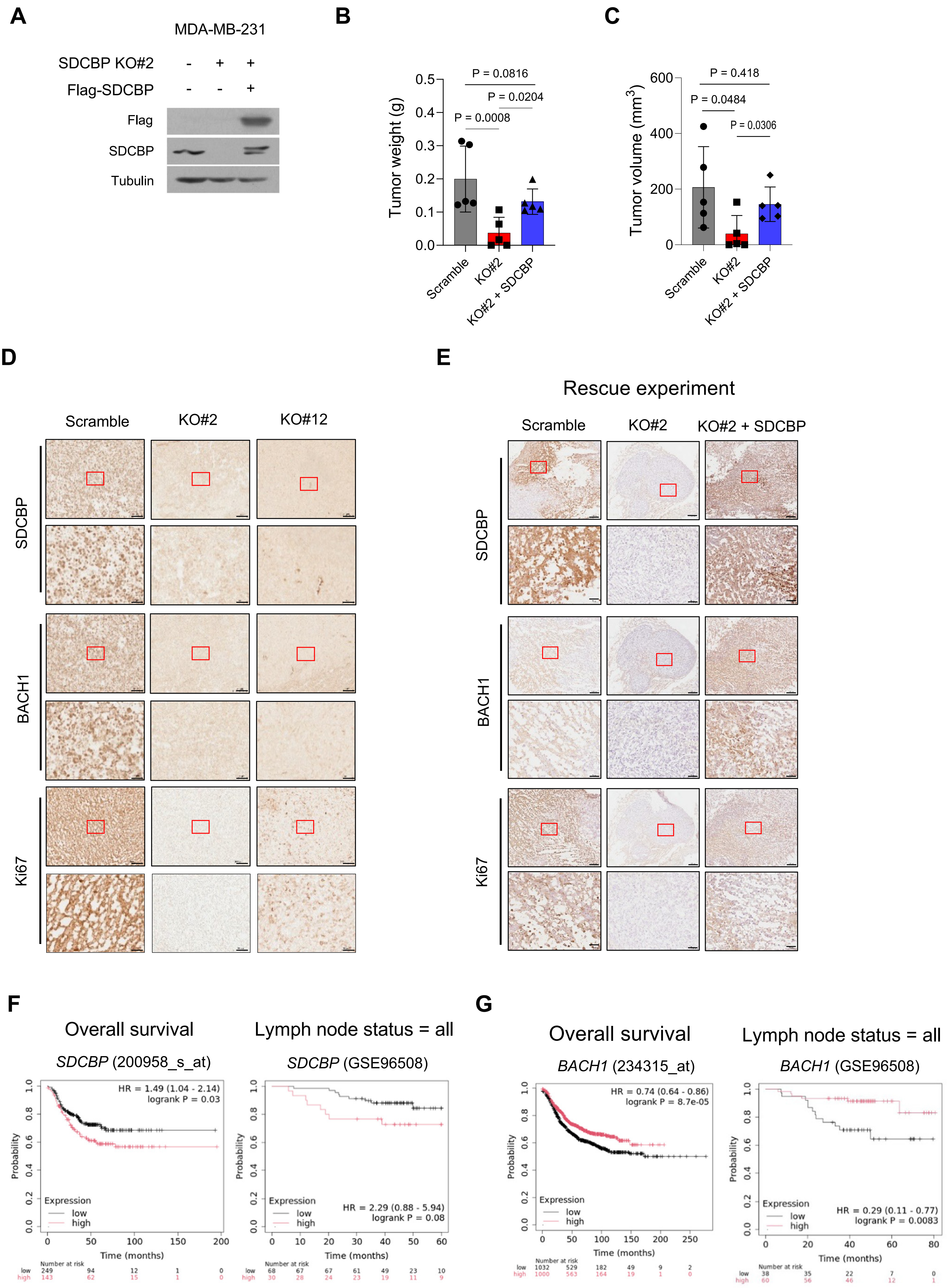
A
MDA-MB-231
SDCBP KO#2 - + +
Flag-SDCBP - - +
Flag
SDCBP
Tubulin
B
Tumor weight (g)
P = 0.0816
P = 0.0204
P = 0.0008
Scramble
KO#2
KO#2 + SDCBP
C
Tumor volume (mm3)
P = 0.418
P = 0.0484
P = 0.0306
Scramble
KO#2
KO#2 + SDCBP
D
Scramble
KO#2
KO#12
SDCBP
BACH1
Ki67
E
Rescue experiment
Scramble
KO#2
KO#2 + SDCBP
SDCBP
BACH1
Ki67
F
Overall survival
SDCBP (200958_s_at)
HR = 1.49 (1.04 - 2.14)
logrank P = 0.03
Lymph node status = all
SDCBP (GSE96508)
HR = 2.29 (0.88 - 5.94)
logrank P = 0.08
Probability
Time (months)
Expression
low
high
Number at risk
G
Overall survival
BACH1 (234315_at)
HR = 0.74 (0.64 - 0.86)
logrank P = 8.7e-05
Lymph node status = all
BACH1 (GSE96508)
HR = 0.29 (0.11 - 0.77)
logrank P = 0.0083
Probability
Time (months)
Expression
low
high
Number at risk

© The Author(s)

Figure EV3. SDCBP promotes tumor growth by upregulating BACH1 and is associated with the survival of TNBC patients.

(**A**) Western blot showing the expression of SDCBP and Flag-SDCBP in Fig. 2O. (**B**) Tumor weights in Fig. 2O ($n = 5$ mice/group). (**C**) Tumor volume in Fig. 2O ($n = 5$ mice/group). (**D**) Representative images of immunohistochemistry staining against SDCBP, BACH1, and Ki67 protein for xenografted tumors isolated from athymic BALB/c nude mice 6 weeks after mammary fat-pad injection of the scramble control or SDCBP-KO MDA-MB-231 cells (1×10^5 cells/mouse; $n = 7$ mice/group). Representative images of IHC staining are shown. Scale bar = 200 µm (upper) and 50 µm (lower), respectively. (**E**) Representative images of immunohistochemistry staining against SDCBP, BACH1, and Ki67 protein for xenografted tumors isolated from athymic BALB/c nude mice 25 days after mammary fat-pad injection of the scramble control, SDCBP-KO MDA-MB-231 cells, or SDCBP-KO MDA-MB-231 cells stably transfected with Flag-SDCBP (1×10^5 cells/mouse; $n = 5$ mice/group). Scale bar = 200 µm (upper) and 50 µm (lower), respectively. (**F**) TCGA data analysis showing association between *SDCBP* mRNA expression and overall survival ($n = 392$) and lymph node status ($n = 98$) of TNBC patients. (**G**) TCGA data analysis showing association between *BACH1* mRNA expression and overall survival ($n = 2032$) and lymph node status ($n = 98$) of TNBC patients. Data are expressed as the mean ± SEM and analyzed using two-way ANOVA (**B**) or two-tailed Student's *t* test (**C**). *P* values less than 0.05 were considered statistically significant. All experiments were repeated at least three times unless otherwise indicated.

 © The Author(s)

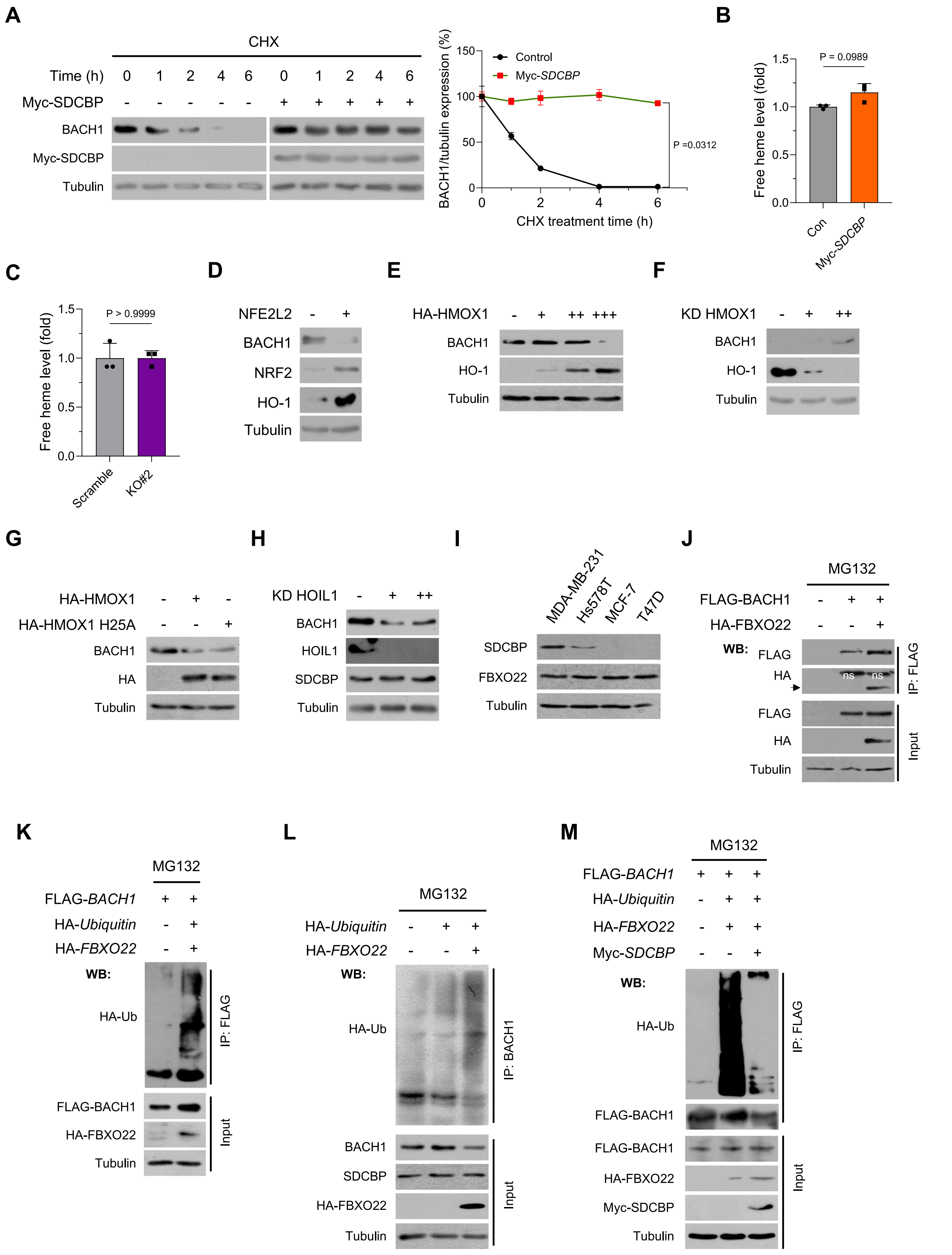
A
CHX
Time (h) 0 1 2 4 6 0 1 2 4 6
Myc-SDCBP - - - - - + + + + +
BACH1
Myc-SDCBP
Tubulin
BACH1/tubulin expression (%)
Control
Myc-SDCBP
P =0.0312
CHX treatment time (h)
B
P = 0.0989
Free heme level (fold)
Con
Myc-SDCBP
C
P > 0.9999
Free heme level (fold)
Scramble
KO#2
D
NFE2L2 - +
BACH1
NRF2
HO-1
Tubulin
E
HA-HMOX1 - + ++ +++
BACH1
HO-1
Tubulin
F
KD HMOX1 - + ++
BACH1
HO-1
Tubulin
G
HA-HMOX1 - + -
HA-HMOX1 H25A - - +
BACH1
HA
Tubulin
H
KD HOIL1 - + ++
BACH1
HOIL1
SDCBP
Tubulin
I
MDA-MB-231
Hs578T
MCF-7
T47D
SDCBP
FBXO22
Tubulin
J
MG132
FLAG-BACH1 - + +
HA-FBXO22 - - +
WB: FLAG
HA
ns
IP: FLAG
FLAG
HA
Tubulin
Input
K
MG132
FLAG-BACH1 + +
HA-Ubiquitin - +
HA-FBXO22 - +
WB:
HA-Ub
IP: FLAG
FLAG-BACH1
HA-FBXO22
Tubulin
Input
L
MG132
HA-Ubiquitin - + +
HA-FBXO22 - - +
WB:
HA-Ub
IP: BACH1
BACH1
SDCBP
HA-FBXO22
Tubulin
Input
M
MG132
FLAG-BACH1 + + +
HA-Ubiquitin - + +
HA-FBXO22 - + +
Myc-SDCBP - - +
WB:
HA-Ub
FLAG-BACH1
IP: FLAG
FLAG-BACH1
HA-FBXO22
Myc-SDCBP
Tubulin
Input

Figure EV4. SDCBP induces BACH1 stability by impairing FBXO22-mediated BACH1 polyubiquitination via an alternative Heme/HO-1-independent mechanism.

(**A**) Left, Western blot showing BACH1 protein expression in MDA-MB-231 cells transfected with control vector or Myc-SDCBP-expressing vector in the presence of CHX protein synthesis inhibitor at various time points. Right, quantification of BACH1 protein levels using densitometry ($n = 3$). (**B**) Free heme level in Hs578T cells transfected with control vector or Myc-SDCBP-expressing vector ($n = 3$). (**C**) Free heme level in scramble and in SDCBP-KO MDA-MB-231 cells ($n = 3$). (**D**) Western blot showing BACH1 protein expression in MDA-MB-231 cells transfected with control vector or NRF2 (encoded by *NFE2L2*)-expressing vector. HO-1 protein expression was considered as the positive control. (**E**) Western blot showing BACH1 protein expression in MDA-MB-231 cells transfected with a control or a HO-1 (encoded by *HMOX1*)-expressing vector. (**F**) Western blot showing BACH1 protein expression in Hs578T cells transfected with scramble or HO-1 siRNA. (**G**) Western blot showing BACH1 protein expression in MDA-MB-231 cells transfected with the HO-1 or the catalytic inactive HO-1 mutant (H25A) plasmid. (**H**) Western blot showing BACH1 and SDCBP protein expression in MDA-MB-231 cells transfected with scramble or HOIL1 siRNA. (**I**) Western blot showing endogenous FBXO22 protein expression in several breast cancer cells. (**J**) Immunoprecipitation showing the interaction of BACH1 with FBXO22 in HEK293 cells transfected with the indicated plasmids. An arrow indicates the specific signal for HA-FBXO22. ns: none specific. (**K**) In vivo ubiquitylation assay showing the increase in the polyubiquitylation of BACH1 by FBXO22 overexpression in HEK293 cells transfected with the indicated plasmids. (**L**) In vivo ubiquitylation assay showing the increase in the polyubiquitylation of BACH1 by FBXO22 overexpression in MDA-MB-231 cells transfected with the indicated plasmids. (**M**) In vivo ubiquitylation assay showing the decrease in FBXO22-mediated polyubiquitylation of BACH1 by SDCBP overexpression in HEK293 cells transfected with the indicated plasmids. Data are expressed as the mean ± SEM and analyzed using two-tailed Student's *t* test with Welch's correction (**A–C**). All experiments were repeated at least three times unless otherwise indicated. *P* values less than 0.05 were considered statistically significant.

 © The Author(s)

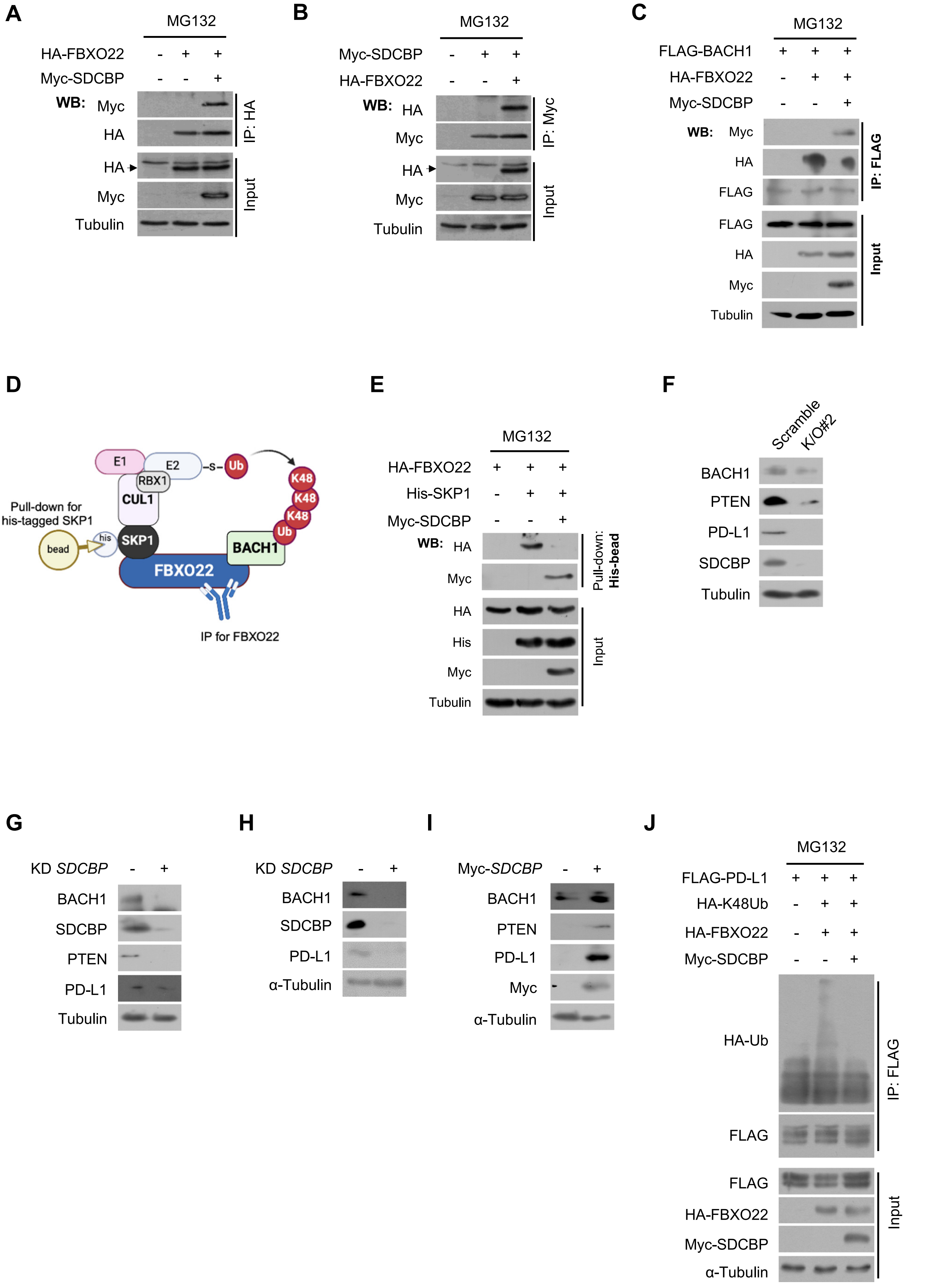
A
MG132
HA-FBXO22 - + +
Myc-SDCBP - - +
WB: Myc
HA
IP: HA
HA
Myc
Tubulin
Input
B
MG132
Myc-SDCBP - + +
HA-FBXO22 - - +
WB: HA
Myc
IP: Myc
HA
Myc
Tubulin
Input
C
MG132
FLAG-BACH1 + + +
HA-FBXO22 - + +
Myc-SDCBP - - +
WB: Myc
HA
FLAG
IP: FLAG
FLAG
HA
Myc
Tubulin
Input
D
E1
E2
-S-
Ub
RBX1
CUL1
K48
K48
K48
Ub
Pull-down for
his-tagged SKP1
bead
his
SKP1
BACH1
FBXO22
IP for FBXO22
E
MG132
HA-FBXO22 + + +
His-SKP1 - + +
Myc-SDCBP - - +
WB: HA
Myc
Pull-down:
His-bead
HA
His
Myc
Tubulin
Input
F
Scramble
K/O#2
BACH1
PTEN
PD-L1
SDCBP
Tubulin
G
KD *SDCBP* - +
BACH1
SDCBP
PTEN
PD-L1
Tubulin
H
KD *SDCBP* - +
BACH1
SDCBP
PD-L1
α-Tubulin
I
Myc-*SDCBP* - +
BACH1
PTEN
PD-L1
Myc
α-Tubulin
J
MG132
FLAG-PD-L1 + + +
HA-K48Ub - + +
HA-FBXO22 - + +
Myc-SDCBP - - +
HA-Ub
FLAG
IP: FLAG
FLAG
HA-FBXO22
Myc-SDCBP
α-Tubulin
Input

© The Author(s)

Figure EV5. SDCBP associates with FBXO22 and impairs SCF^{FBXO22}-targeted substrates for K48-linked degradative ubiquitination.

(**A**) Immunoprecipitation showing the interaction of FBXO22 with SDCBP in HEK293 cells transfected with the indicated plasmids. An arrow indicates the specific signal for HA-FBXO22. (**B**) Immunoprecipitation showing the interaction of SDCBP with FBXO22 in HEK293 cells transfected with the indicated plasmids. An arrow indicates the specific signal for HA-FBXO22. (**C**) Co-immunoprecipitation showing the interaction of FBXO22 with BACH1 in HEK293 cells with or without SDCBP after the indicated transfections. (**D**) Schematic of experimental design to investigate the assembly of SCF^{FBXO22}–BACH1 complex via His Pull-down assay and endogenous IP assay in Fig. 4D–G. (**E**) His-pulldown assay showing the interaction of FBXO22 with SKP1 in HEK293 cells with control vector or Myc-SDCBP-expressing vector after the indicated transfections. See also Appendix Fig. S1A. (**F**) Western blot showing BACH1, PTEN, and PD-L1 protein expression in scramble and in SDCBP-KO MDA-MB-231 cells. (**G**) Western blot showing BACH1, PTEN, and PD-L1 protein expression in A549 cells transfected with scramble or SDCBP siRNA. (**H**) Western blot showing BACH1 and PD-L1 protein expression in NCI-H1299 cells transfected with scramble or SDCBP siRNA. (**I**) Western blot showing BACH1, PTEN, and PD-L1 protein expression in Hs578T cells transfected with control vector or Myc-SDCBP-expressing vector. (**J**) In vivo ubiquitylation assay showing the inhibitory effect of SDCBP on SCF^{FBXO22}-mediated K48-linked polyubiquitylation of BACH1 in HEK293 cells transfected with the indicated plasmids.

 © The Author(s)
