# Supplementary material for: SDCBP/Syntenin-1 stabilizes BACH1 by disassembling the SCFFBXO22–BACH1 complex in triple-negative breast cancer
Source: EMBO J. 2025 Apr 22;44(11):3085–120. doi: 10.1038/s44318-025-00440-1 (PMC12130529; doi:10.1038/s44318-025-00440-1)
Supplement: Supplementary file 3 — Source data Fig. 1 [file 44318_2025_440_MOESM3_ESM.zip › 20250321_EMBOJ-2024-118617R_Figure 1/EMBOJ-2024-118617_Figure 1L.zip!/EMBOJ-2024-118617_Figure 1L/EMBOJ-2024-118617_Figure 1L.pdf]

Fig 1L

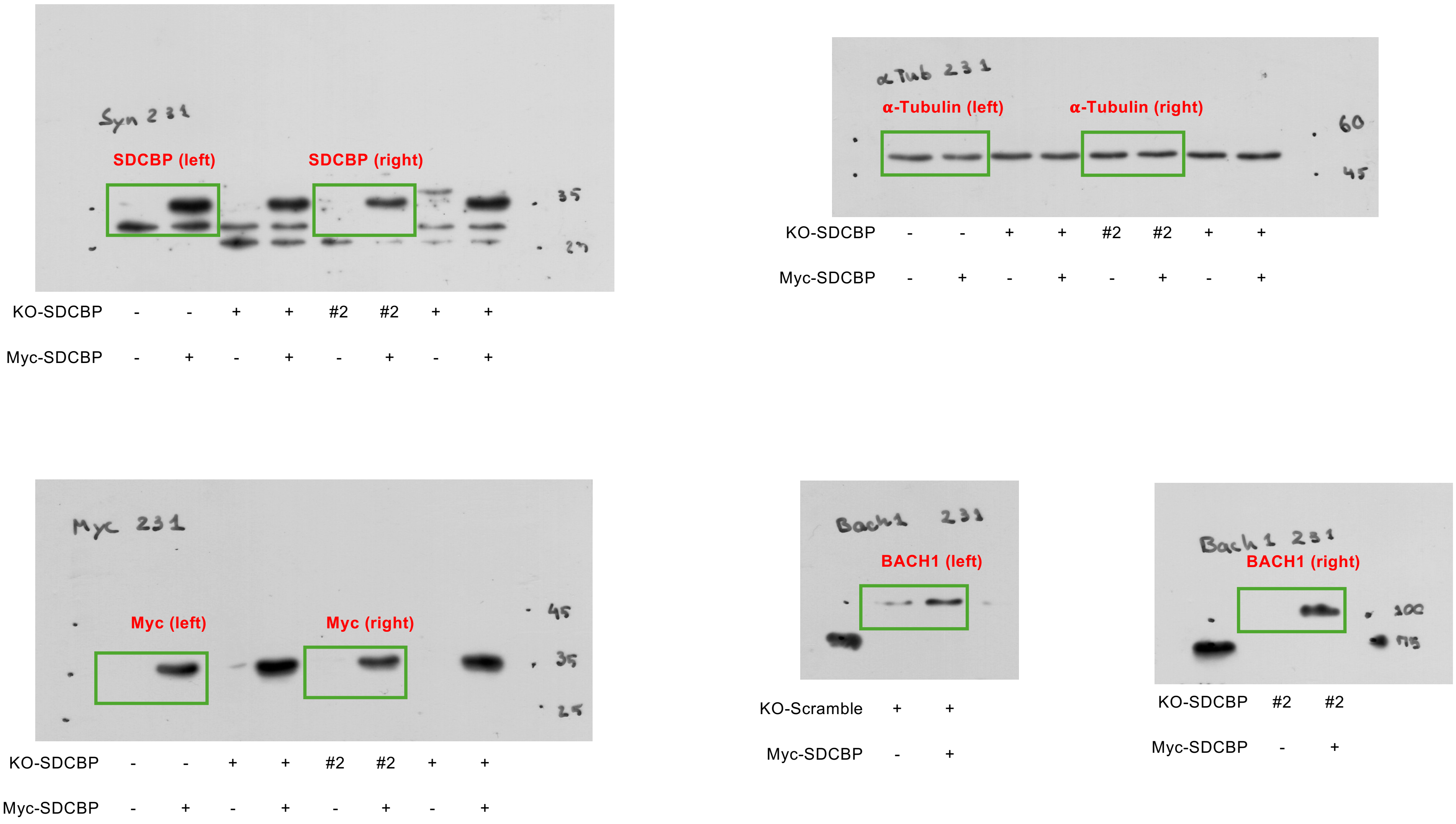

Syn 231
SDCBP (left)
SDCBP (right)
35
KO-SDCBP - - + + #2 #2 + +
Myc-SDCBP - + - + - + - +
αTub 231
α-Tubulin (left)
α-Tubulin (right)
60
45
KO-SDCBP - - + + #2 #2 + +
Myc-SDCBP - + - + - + - +
Myc 231
Myc (left)
Myc (right)
45
35
25
KO-SDCBP - - + + #2 #2 + +
Myc-SDCBP - + - + - + - +
Bach1 231
BACH1 (left)
KO-Scramble + +
Myc-SDCBP - +
Bach1 231
BACH1 (right)
100
75
KO-SDCBP #2 #2
Myc-SDCBP - +
