# Supplementary material for: SDCBP/Syntenin-1 stabilizes BACH1 by disassembling the SCFFBXO22–BACH1 complex in triple-negative breast cancer
Source: EMBO J. 2025 Apr 22;44(11):3085–120. doi: 10.1038/s44318-025-00440-1 (PMC12130529; doi:10.1038/s44318-025-00440-1)
Supplement: Supplementary file 5 — Source data Fig. 3 [file 44318_2025_440_MOESM5_ESM.zip › 20250321_EMBOJ-2024-118617R_Figure 3/EMBOJ-2024-118617_Figure 3B.zip!/EMBOJ-2024-118617_Figure 3B/EMBOJ-2024-118617_Figure 3B.pdf]

Fig 3B

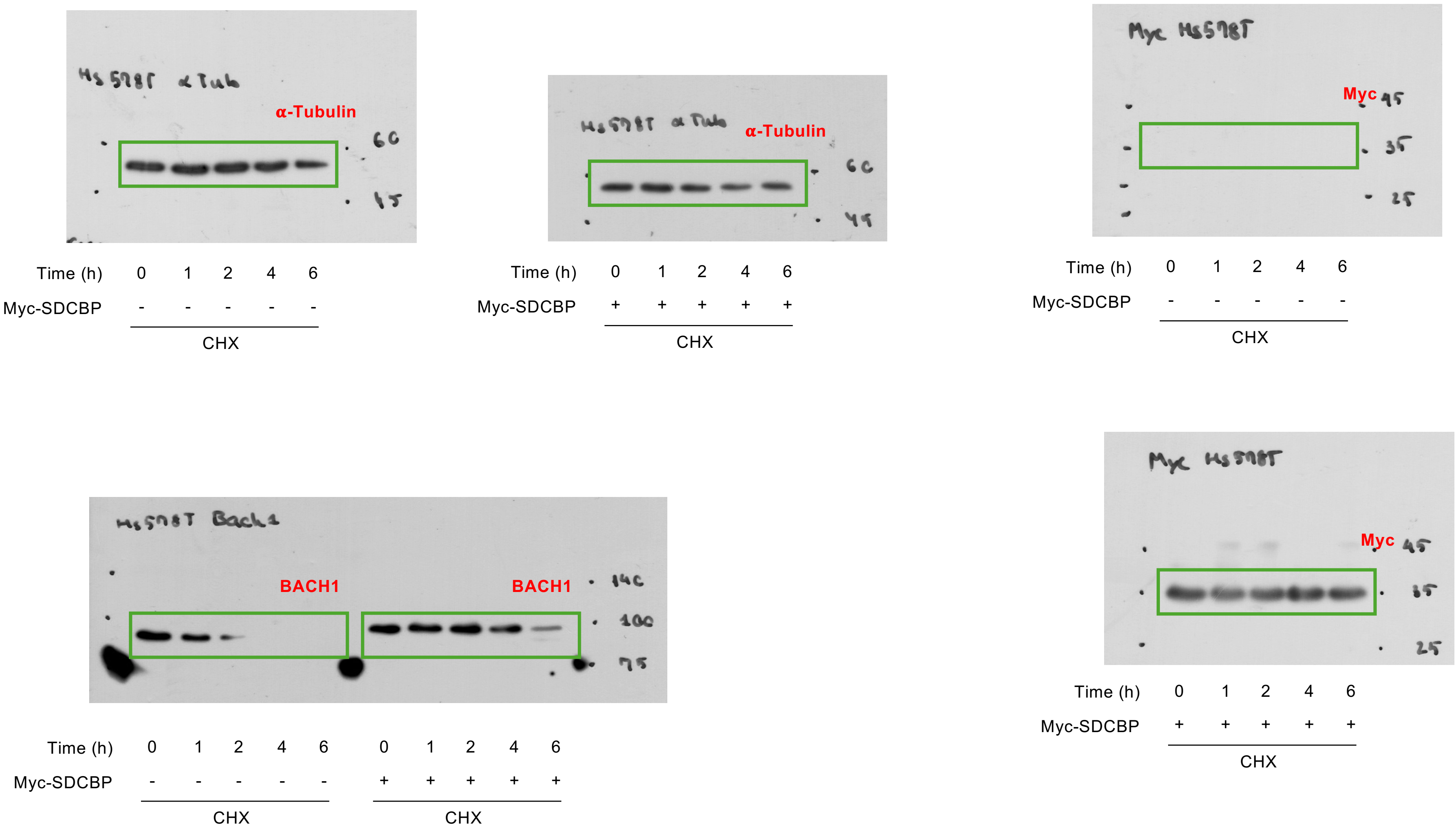
α-Tubulin
Time (h) 0 1 2 4 6
Myc-SDCBP - - - - -
CHX
α-Tubulin
Time (h) 0 1 2 4 6
Myc-SDCBP + + + + +
CHX
Myc
Time (h) 0 1 2 4 6
Myc-SDCBP - - - - -
CHX
BACH1
BACH1
Time (h) 0 1 2 4 6 0 1 2 4 6
Myc-SDCBP - - - - - + + + + +
CHX
CHX
Myc
Time (h) 0 1 2 4 6
Myc-SDCBP + + + + +
CHX
