# Supplementary material for: SDCBP/Syntenin-1 stabilizes BACH1 by disassembling the SCFFBXO22–BACH1 complex in triple-negative breast cancer
Source: EMBO J. 2025 Apr 22;44(11):3085–120. doi: 10.1038/s44318-025-00440-1 (PMC12130529; doi:10.1038/s44318-025-00440-1)
Supplement: Supplementary file 5 — Source data Fig. 3 [file 44318_2025_440_MOESM5_ESM.zip › 20250321_EMBOJ-2024-118617R_Figure 3/EMBOJ-2024-118617_Figure 3C.zip!/EMBOJ-2024-118617_Figure 3C/EMBOJ-2024-118617_Figure 3C.pdf]

Fig 3C

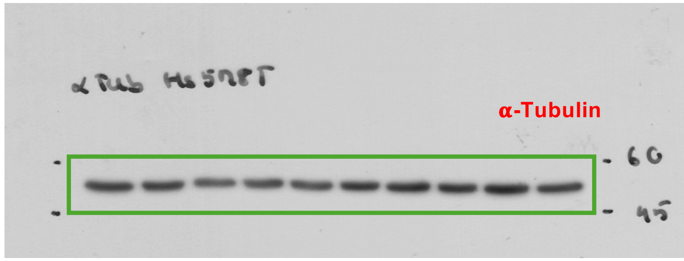

Time (h)	0	1	2	4	6	0	1	2	4	6
KD-FBXO22	-	-	-	-	-	+	+	+	+	+

CHX

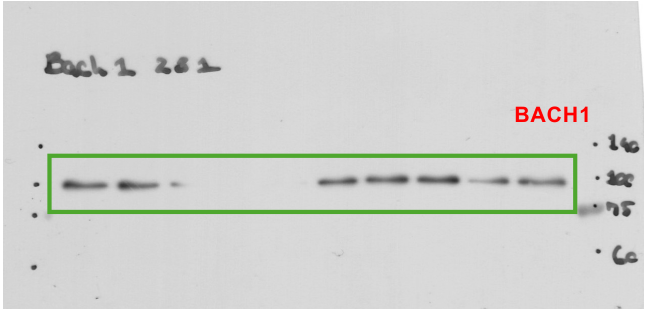

Time (h)	0	1	2	4	6	0	1	2	4	6
KD-FBXO22	-	-	-	-	-	+	+	+	+	+

CHX

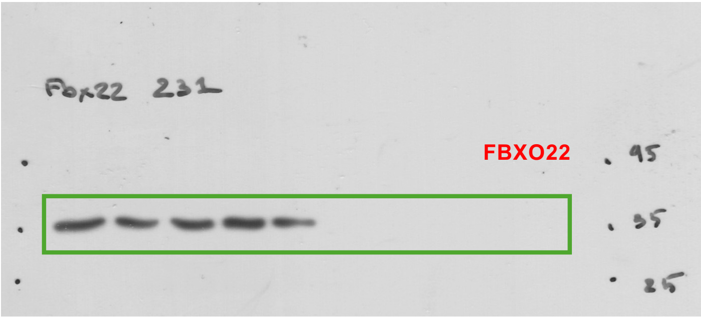

Time (h)	0	1	2	4	6	0	1	2	4	6
KD-FBXO22	-	-	-	-	-	+	+	+	+	+

CHX
