# Supplementary material for: SDCBP/Syntenin-1 stabilizes BACH1 by disassembling the SCFFBXO22–BACH1 complex in triple-negative breast cancer
Source: EMBO J. 2025 Apr 22;44(11):3085–120. doi: 10.1038/s44318-025-00440-1 (PMC12130529; doi:10.1038/s44318-025-00440-1)
Supplement: Supplementary file 5 — Source data Fig. 3 [file 44318_2025_440_MOESM5_ESM.zip › 20250321_EMBOJ-2024-118617R_Figure 3/EMBOJ-2024-118617_Figure 3F.zip!/EMBOJ-2024-118617_Figure 3F/EMBOJ-2024-118617_Figure 3F.pdf]

Fig 3F

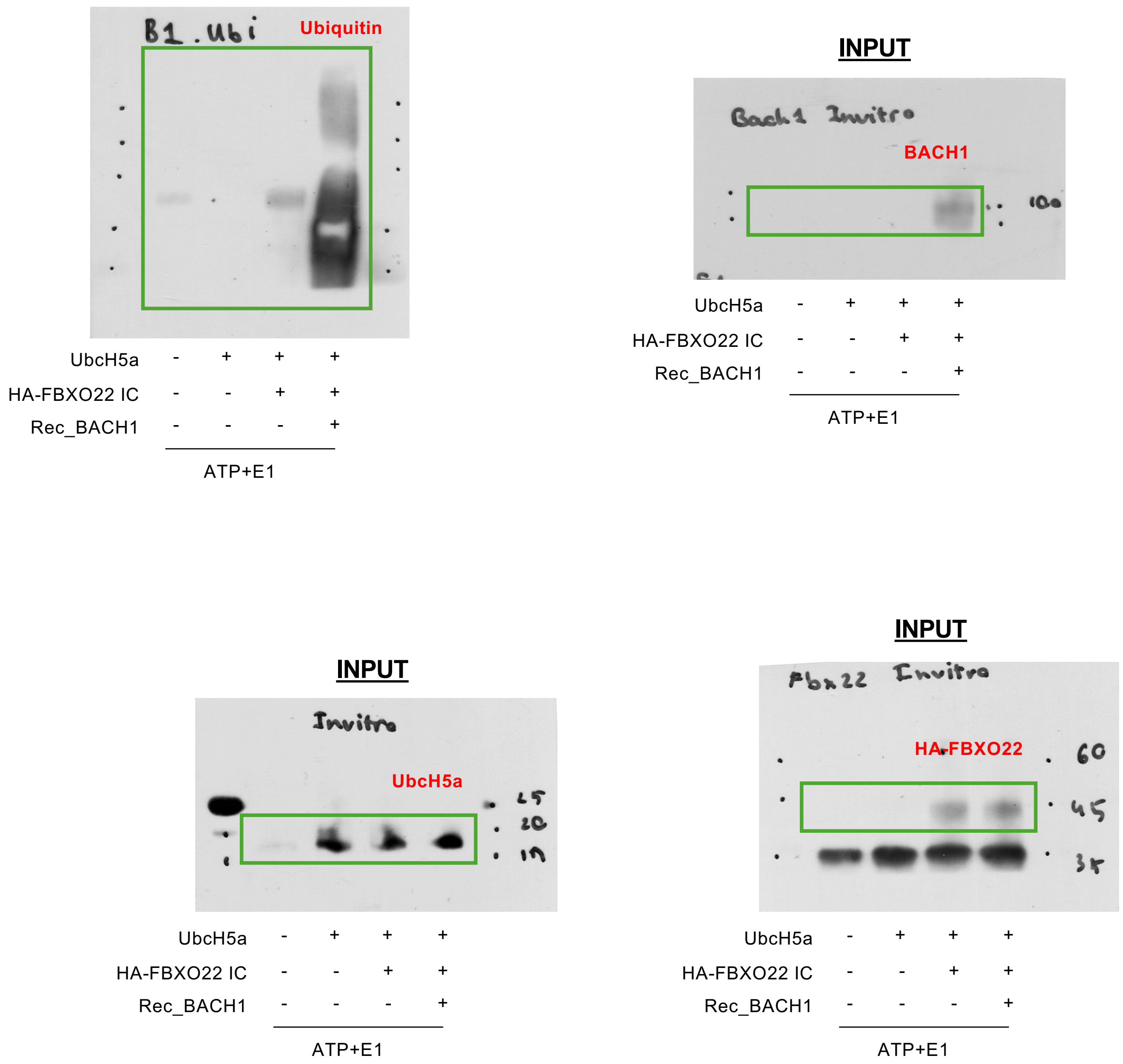
Ubiquitin
UbcH5a - + + +
HA-FBXO22 IC - - + +
Rec_BACH1 - - - +
ATP+E1
INPUT
BACH1
UbcH5a - + + +
HA-FBXO22 IC - - + +
Rec_BACH1 - - - +
ATP+E1
INPUT
UbcH5a
UbcH5a - + + +
HA-FBXO22 IC - - + +
Rec_BACH1 - - - +
ATP+E1
INPUT
HA-FBXO22
UbcH5a - + + +
HA-FBXO22 IC - - + +
Rec_BACH1 - - - +
ATP+E1
