# Supplementary material for: SDCBP/Syntenin-1 stabilizes BACH1 by disassembling the SCFFBXO22–BACH1 complex in triple-negative breast cancer
Source: EMBO J. 2025 Apr 22;44(11):3085–120. doi: 10.1038/s44318-025-00440-1 (PMC12130529; doi:10.1038/s44318-025-00440-1)
Supplement: Supplementary file 6 — Source data Fig. 4 [file 44318_2025_440_MOESM6_ESM.zip › 20250321_EMBOJ-2024-118617R_Figure 4/EMBOJ-2024-118617_Figure 4H.zip!/EMBOJ-2024-118617_Figure 4H/EMBOJ-2024-118617_Figure 4H.pdf]

Fig 4H

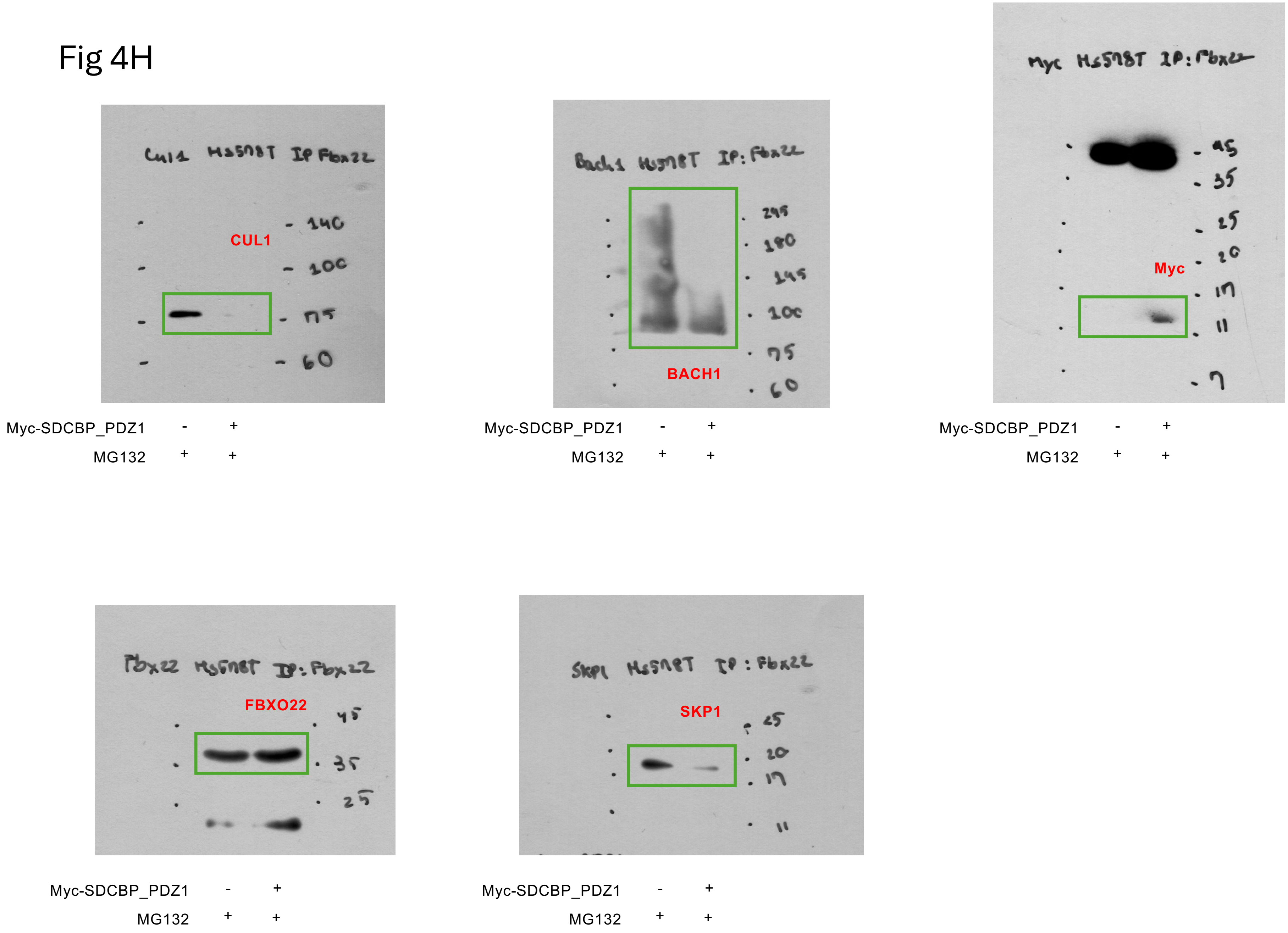
CUL1
140
100
75
60
BACH1
245
180
135
100
75
60
Myc
45
35
25
20
17
11
7
FBXO22
45
35
25
SKP1
25
20
17
11
Myc-SDCBP_PDZ1 - +
MG132 + +

Fig 4H

INPUT

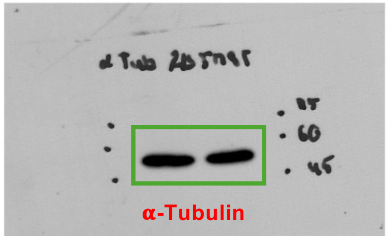

Myc-SDCBP_PDZ1 - +
MG132 + +

INPUT

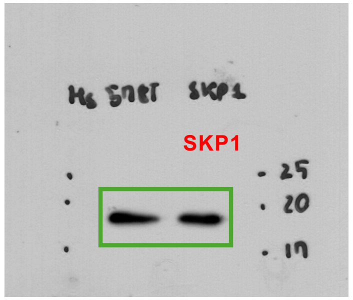

Myc-SDCBP_PDZ1 - +
MG132 + +

INPUT

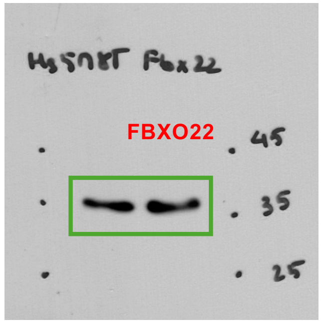

Myc-SDCBP_PDZ1 - +
MG132 + +

INPUT

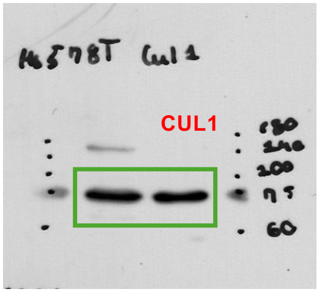

Myc-SDCBP_PDZ1 - +
MG132 + +

INPUT

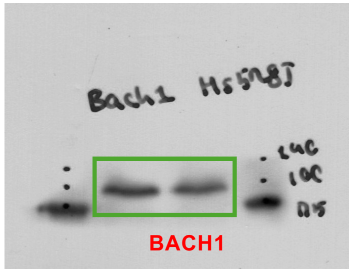

Myc-SDCBP_PDZ1 - +
MG132 + +

INPUT

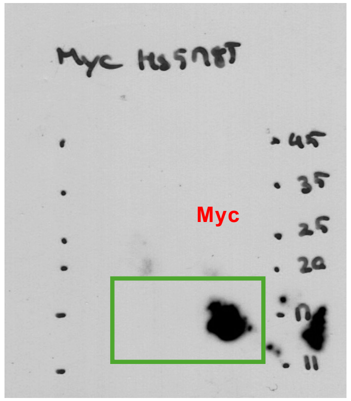

Myc-SDCBP_PDZ1 - +
MG132 + +
