# Supplementary material for: SDCBP/Syntenin-1 stabilizes BACH1 by disassembling the SCFFBXO22–BACH1 complex in triple-negative breast cancer
Source: EMBO J. 2025 Apr 22;44(11):3085–120. doi: 10.1038/s44318-025-00440-1 (PMC12130529; doi:10.1038/s44318-025-00440-1)
Supplement: Supplementary file 6 — Source data Fig. 4 [file 44318_2025_440_MOESM6_ESM.zip › 20250321_EMBOJ-2024-118617R_Figure 4/EMBOJ-2024-118617_Figure 4E.zip!/EMBOJ-2024-118617_Figure 4E/EMBOJ-2024-118617_Figure 4E.pdf]

Fig 4E

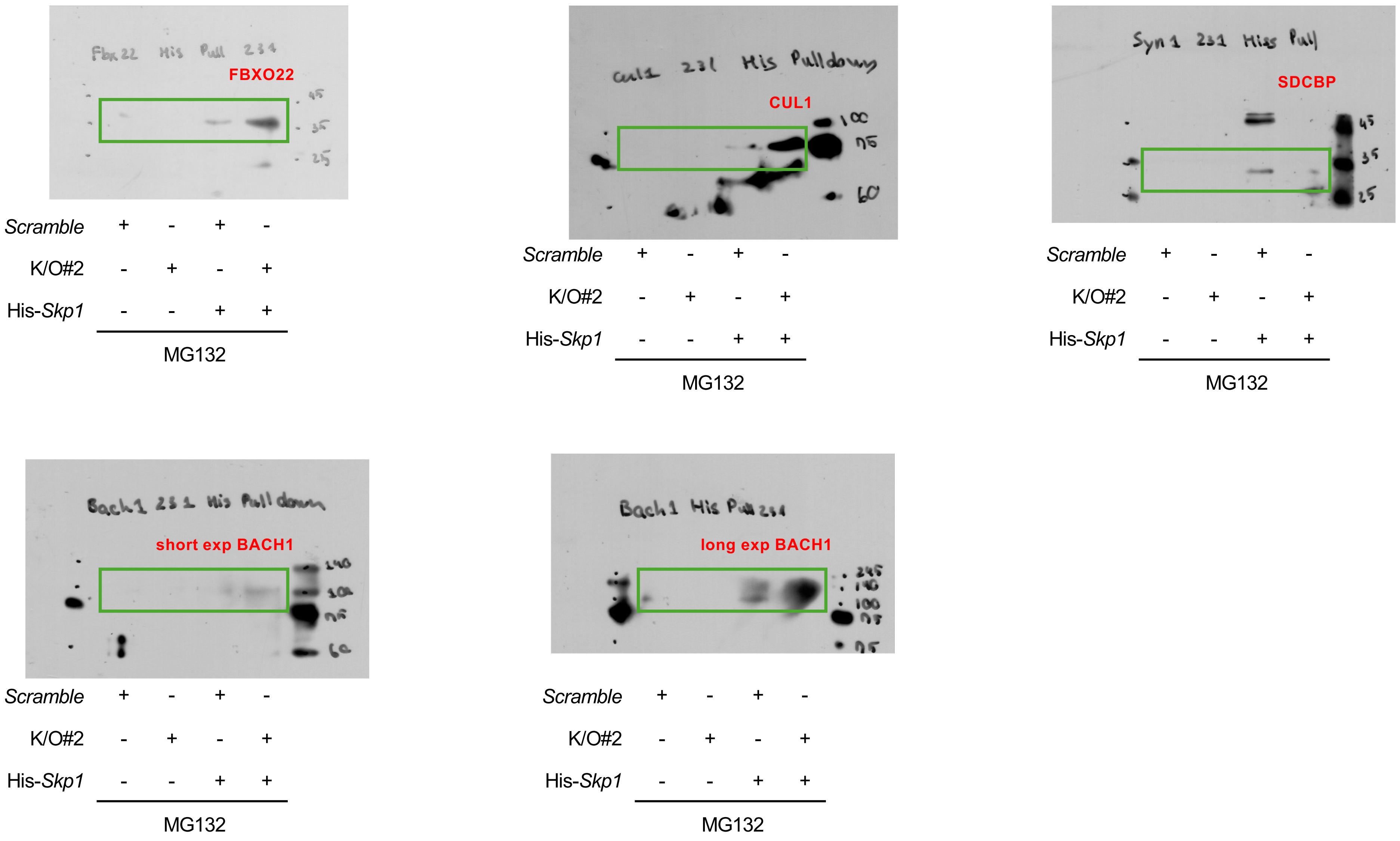
FBXO22
Scramble + - + -
K/O#2 - + - +
His-Skp1 - - + +
MG132
CUL1
Scramble + - + -
K/O#2 - + - +
His-Skp1 - - + +
MG132
SDCBP
Scramble + - + -
K/O#2 - + - +
His-Skp1 - - + +
MG132
short exp BACH1
Scramble + - + -
K/O#2 - + - +
His-Skp1 - - + +
MG132
long exp BACH1
Scramble + - + -
K/O#2 - + - +
His-Skp1 - - + +
MG132

Fig 4E

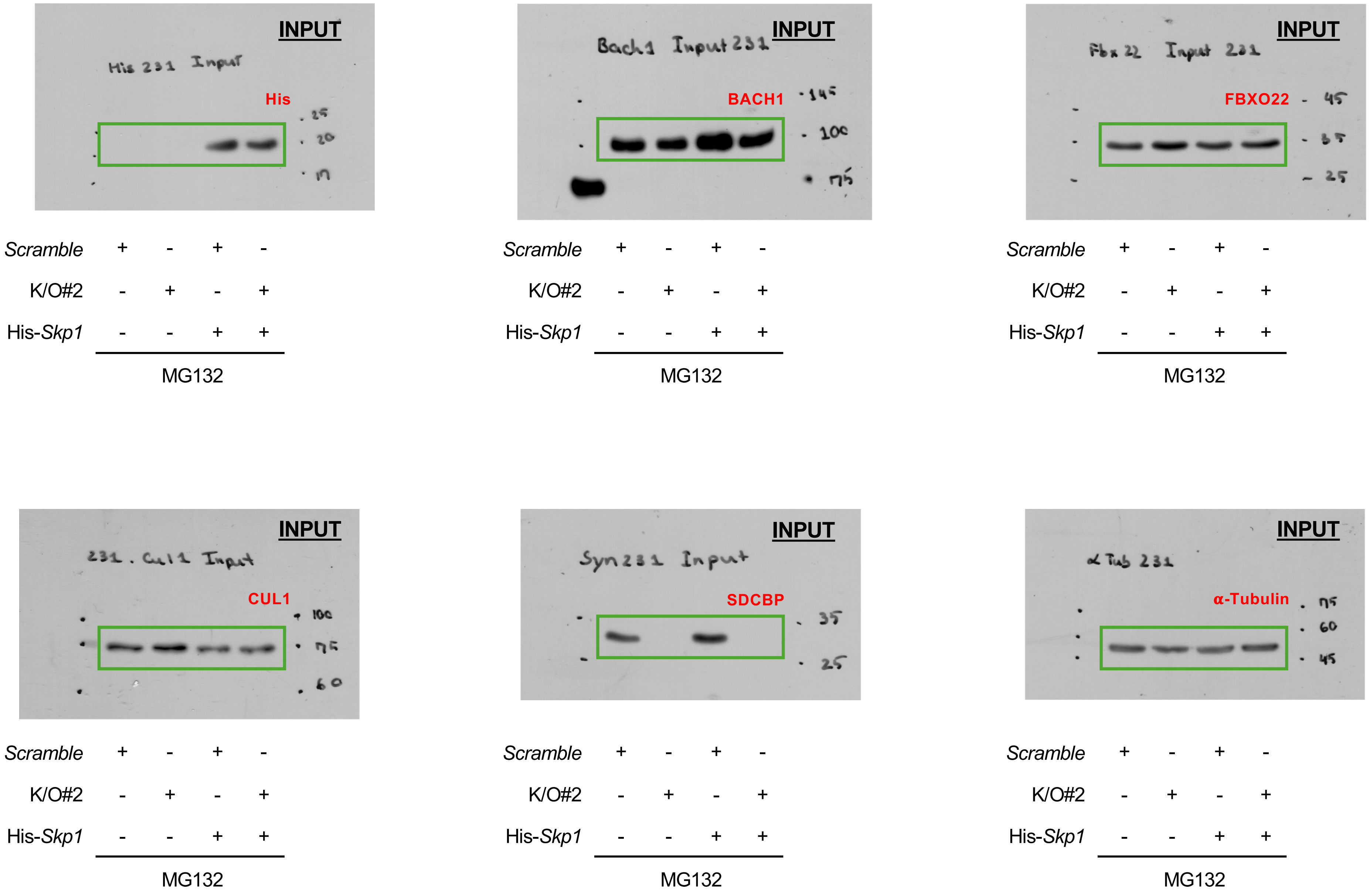
INPUT
His 231 Input
His
25
20
17
Scramble + - + -
K/O#2 - + - +
His-Skp1 - - + +
MG132
INPUT
Bach1 Input 231
BACH1
145
100
75
Scramble + - + -
K/O#2 - + - +
His-Skp1 - - + +
MG132
INPUT
Fbx 22 Input 231
FBXO22
45
35
25
Scramble + - + -
K/O#2 - + - +
His-Skp1 - - + +
MG132
INPUT
231. Cul1 Input
CUL1
100
75
60
Scramble + - + -
K/O#2 - + - +
His-Skp1 - - + +
MG132
INPUT
Syn 231 Input
SDCBP
35
25
Scramble + - + -
K/O#2 - + - +
His-Skp1 - - + +
MG132
INPUT
α Tub 231
α-Tubulin
75
60
45
Scramble + - + -
K/O#2 - + - +
His-Skp1 - - + +
MG132
