# Supplementary material for: SDCBP/Syntenin-1 stabilizes BACH1 by disassembling the SCFFBXO22–BACH1 complex in triple-negative breast cancer
Source: EMBO J. 2025 Apr 22;44(11):3085–120. doi: 10.1038/s44318-025-00440-1 (PMC12130529; doi:10.1038/s44318-025-00440-1)
Supplement: Supplementary file 6 — Source data Fig. 4 [file 44318_2025_440_MOESM6_ESM.zip › 20250321_EMBOJ-2024-118617R_Figure 4/EMBOJ-2024-118617_Figure 4G.zip!/EMBOJ-2024-118617_Figure 4G/EMBOJ-2024-118617_Figure 4G.pdf]

Fig 4G

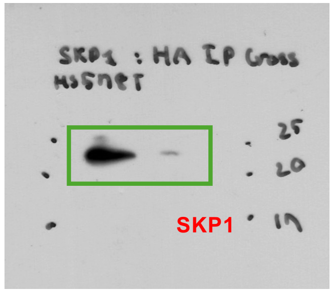

Myc-SDCBP	-	+
MG132	+	+

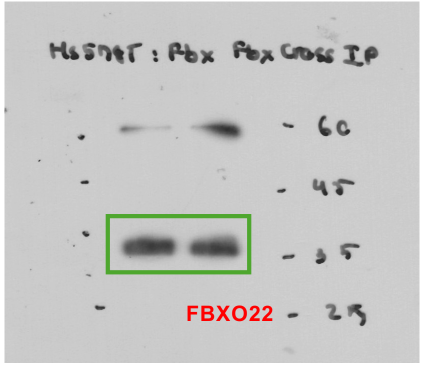

Myc-SDCBP	-	+
MG132	+	+

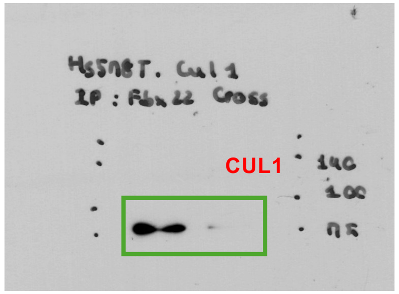

Myc-SDCBP	-	+
MG132	+	+

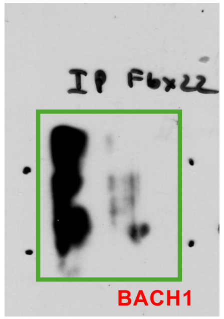

Myc-SDCBP	-	+
MG132	+	+

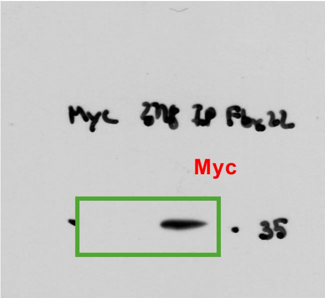

Myc-SDCBP	-	+
MG132	+	+

Fig 4G

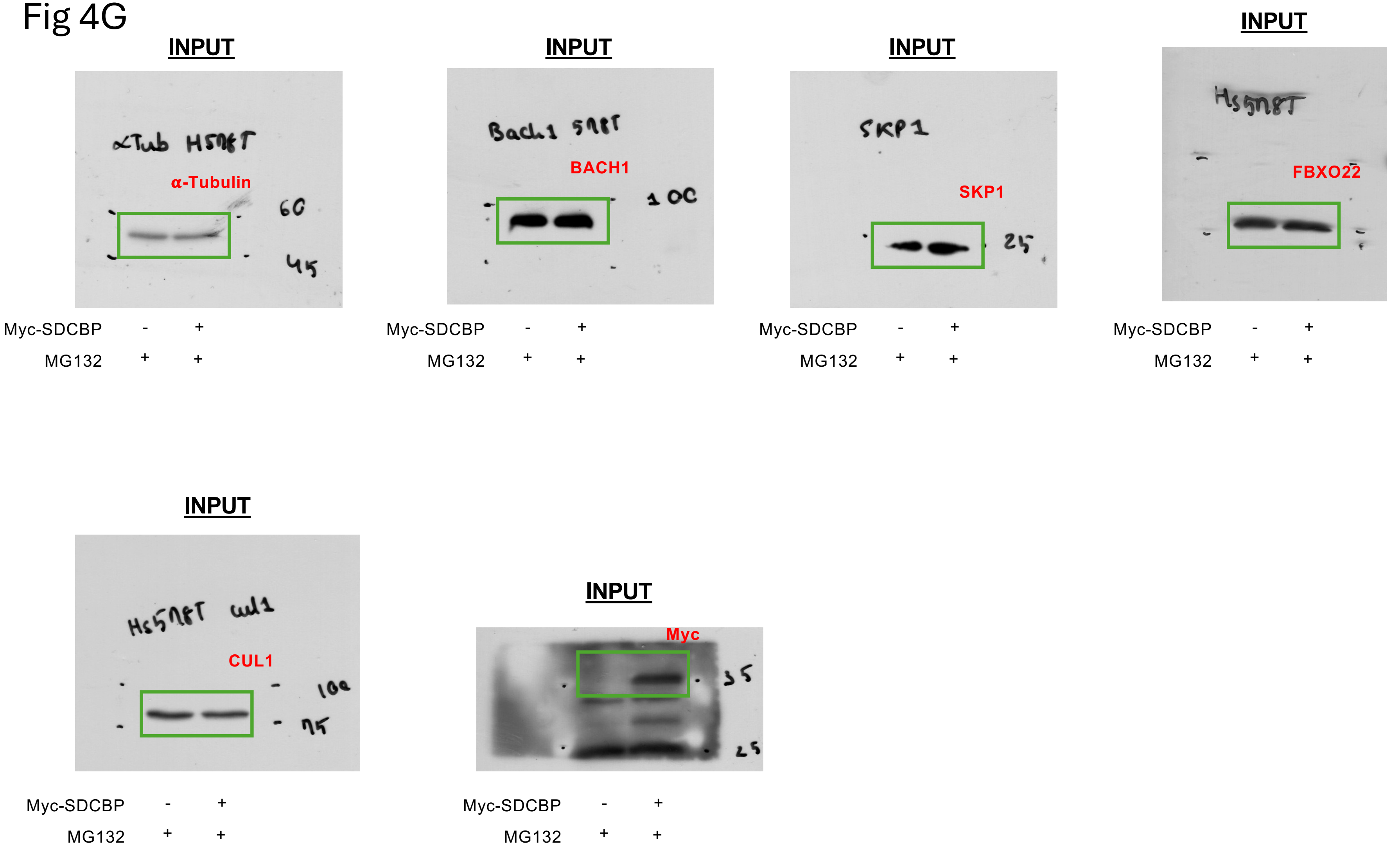
INPUT
α-Tubulin
Myc-SDCBP - +
MG132 + +
INPUT
BACH1
Myc-SDCBP - +
MG132 + +
INPUT
SKP1
Myc-SDCBP - +
MG132 + +
INPUT
FBXO22
Myc-SDCBP - +
MG132 + +
INPUT
CUL1
Myc-SDCBP - +
MG132 + +
INPUT
Myc
Myc-SDCBP - +
MG132 + +
