# Supplementary material for: SDCBP/Syntenin-1 stabilizes BACH1 by disassembling the SCFFBXO22–BACH1 complex in triple-negative breast cancer
Source: EMBO J. 2025 Apr 22;44(11):3085–120. doi: 10.1038/s44318-025-00440-1 (PMC12130529; doi:10.1038/s44318-025-00440-1)
Supplement: Supplementary file 6 — Source data Fig. 4 [file 44318_2025_440_MOESM6_ESM.zip › 20250321_EMBOJ-2024-118617R_Figure 4/EMBOJ-2024-118617_Figure 4A.zip!/EMBOJ-2024-118617_Figure 4A/EMBOJ-2024-118617_Figure 4A.pdf]

Fig 4A

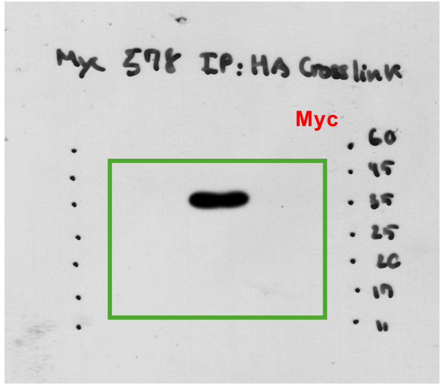

Myc-SDCBP	-	+	-
Myc-SDCBP_△4	-	-	+

INPUT

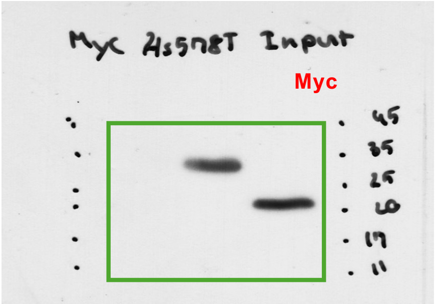

Myc-SDCBP	-	+	-
Myc-SDCBP_△4	-	-	+

INPUT

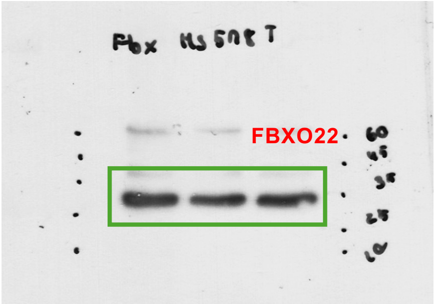

Myc-SDCBP	-	+	-
Myc-SDCBP_△4	-	-	+

INPUT

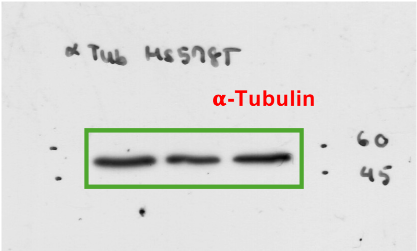

Myc-SDCBP	-	+	-
Myc-SDCBP_△4	-	-	+
