# Supplementary material for: SDCBP/Syntenin-1 stabilizes BACH1 by disassembling the SCFFBXO22–BACH1 complex in triple-negative breast cancer
Source: EMBO J. 2025 Apr 22;44(11):3085–120. doi: 10.1038/s44318-025-00440-1 (PMC12130529; doi:10.1038/s44318-025-00440-1)
Supplement: Supplementary file 7 — Source data Fig. 5 [file 44318_2025_440_MOESM7_ESM.zip › 20250321_EMBOJ-2024-118617R_Figure 5/EMBOJ-2024-118617_Figure 5C.zip!/EMBOJ-2024-118617_Figure 5C/EMBOJ-2024-118617_Figure 5C.pdf]

Fig 5B

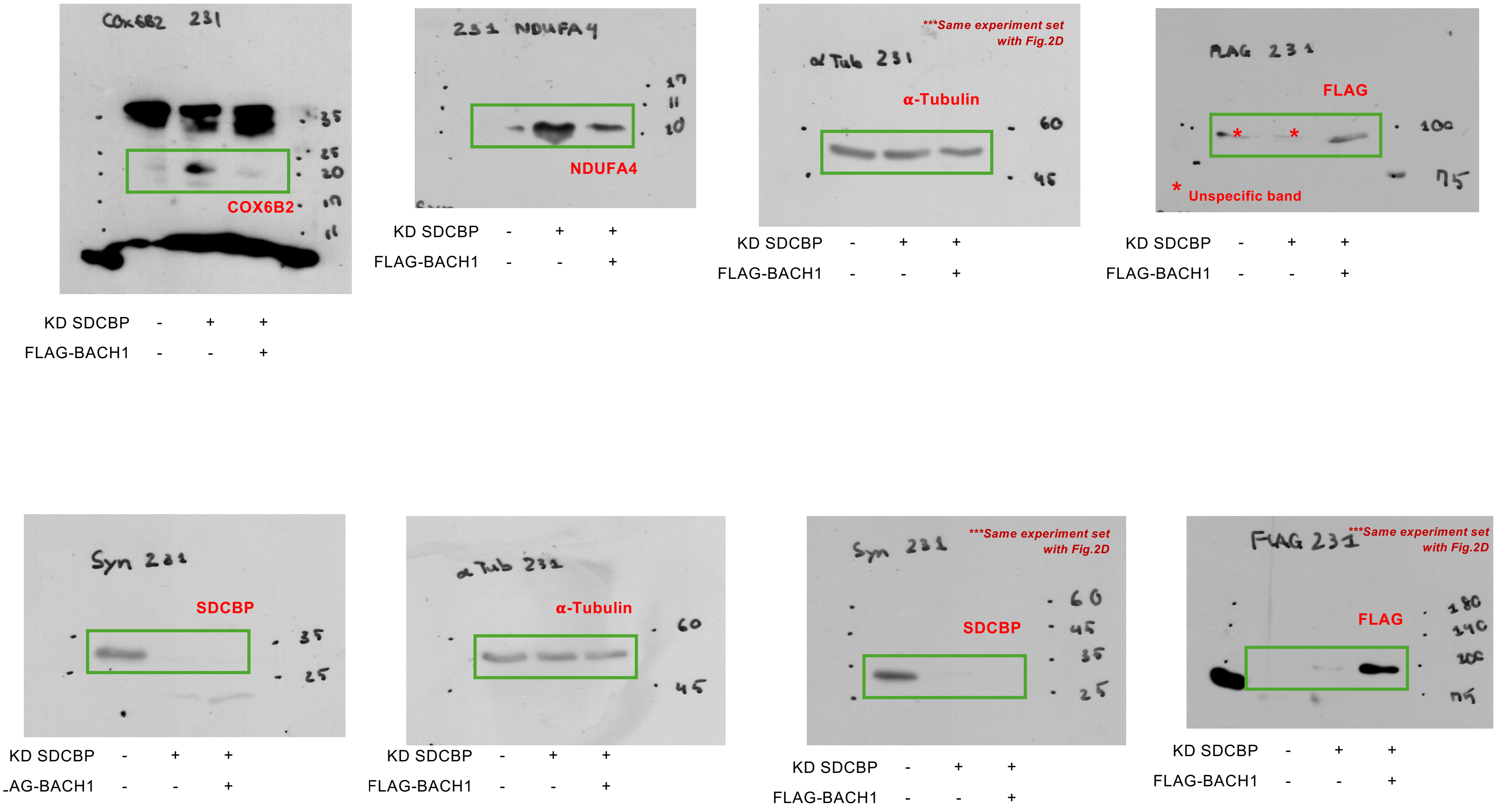
COX6B2
KD SDCBP - + +
FLAG-BACH1 - - +
NDUFA4
KD SDCBP - + +
FLAG-BACH1 - - +
***Same experiment set with Fig.2D
α-Tubulin
KD SDCBP - + +
FLAG-BACH1 - - +
FLAG
* Unspecific band
KD SDCBP - + +
FLAG-BACH1 - - +
SDCBP
KD SDCBP - + +
_AG-BACH1 - - +
α-Tubulin
KD SDCBP - + +
FLAG-BACH1 - - +
***Same experiment set with Fig.2D
SDCBP
KD SDCBP - + +
FLAG-BACH1 - - +
***Same experiment set with Fig.2D
FLAG
KD SDCBP - + +
FLAG-BACH1 - - +
