# Supplementary material for: SDCBP/Syntenin-1 stabilizes BACH1 by disassembling the SCFFBXO22–BACH1 complex in triple-negative breast cancer
Source: EMBO J. 2025 Apr 22;44(11):3085–120. doi: 10.1038/s44318-025-00440-1 (PMC12130529; doi:10.1038/s44318-025-00440-1)
Supplement: Supplementary file 7 — Source data Fig. 5 [file 44318_2025_440_MOESM7_ESM.zip › 20250321_EMBOJ-2024-118617R_Figure 5/EMBOJ-2024-118617_Figure 5G.zip!/EMBOJ-2024-118617_Figure 5G/EMBOJ-2024-118617_Figure 5G.pdf]

Fig 5G

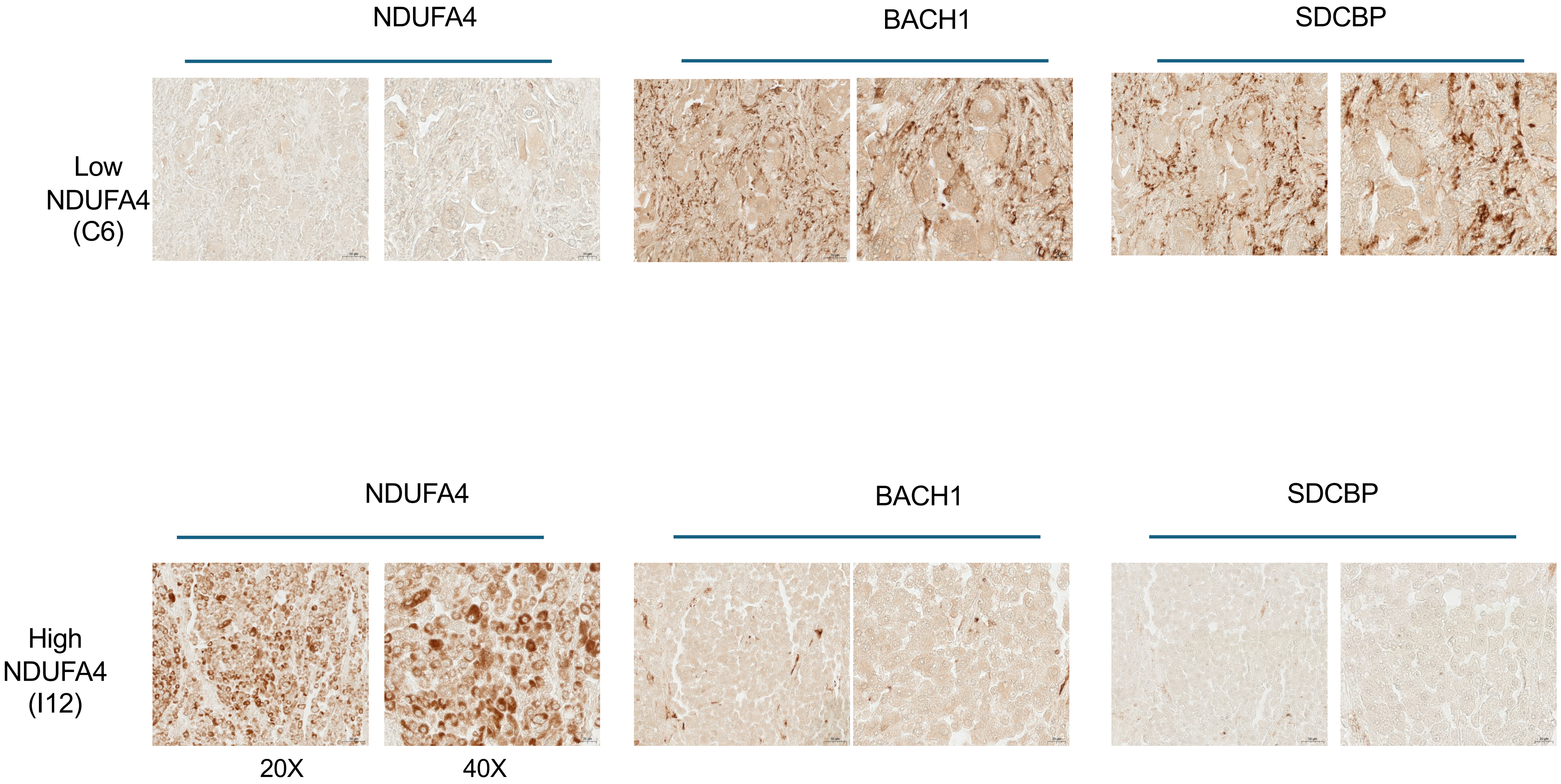

NDUFA4
BACH1
SDCBP
Low NDUFA4 (C6)
NDUFA4
BACH1
SDCBP
High NDUFA4 (I12)
20X
40X
